# Porcupine inhibition is a promising pharmacological treatment for severe sclerosteosis pathologies

**DOI:** 10.1038/s41413-025-00406-3

**Published:** 2025-04-07

**Authors:** Timothy J. Dreyer, Jacob A. C. Keen, Leah M. Wells, Mark Hopkinson, Isabel R. Orriss, Gill Holdsworth, Andrew A. Pitsillides, Scott J. Roberts

**Affiliations:** 1 Skeletal Biology Group, Department of Comparative Biomedical Sciences, The Royal Veterinary College, London, UK; 2 UCB Pharma, Slough, UK

**Keywords:** Endocrine system and metabolic diseases, Bone, Metabolic bone disease

## Abstract

Sclerosteosis, an ultra-rare disorder characterised by high bone mass (HBM) and skeletal overgrowth, leads to facial paralysis, hearing loss and raised intracranial pressure, which is currently managed only through high-risk surgery. Sclerosteosis is caused by *SOST* mutations and loss of functional sclerostin, a protein that suppresses osteogenesis by antagonising Wnt/β-catenin signalling. Herein, using in vitro and in vivo approaches, we explore whether LGK974, another potent Wnt inhibitor that targets porcupine (PORCN, Wnt-specific acyltransferase), is a promising sclerosteosis therapeutic. In vitro assays showed that 100 nmol/L LGK974 significantly reduced osteoblast alkaline phosphatase (ALP) activity/mineralisation, decreased Wnt/osteoblast marker (*Axin2*, *Runx2* and *Ocn*) expression, and downregulated ossification and the Wnt signalling pathway, without affecting osteoclast numbers/resorption. To assess in vivo effects, 6-week-old male and female *Sost* deficient (*Sost*^-/-^) mice received LGK974 for 4 weeks and right hindlimbs were subjected to 20 N peak loading to assess mechanoadaptive interactions. µCT revealed significant reductions in vertebral trabecular number and lower cortical bone volume in loaded and non-loaded tibiae in male and female LGK974-treated *Sost*^-/-^ mice. Interestingly, the target engagement biomarker Axin2 was only significantly reduced in male vertebrae, which may indicate differences in male and female response to LGK974. This study also shows that PORCN inhibition may effectively limit characteristic HBM and skeletal overgrowth in sclerosteosis patients at sites with severe pathology.

## Introduction

Disruption of the fine balance between bone resorption and formation can generate high bone mass (HBM) disorders, with severe symptoms.^1,2^ Sclerosteosis (OMIM accession number 269500) is an ultra-rare (~100 cases), extreme HBM condition found predominantly in South African Afrikaners.^3–20^ Sclerosteosis arises via nonsense, missense or frameshift *SOST* gene mutations that alter expression and processing of sclerostin, the *SOST* gene transcript, resulting in loss of protein function.^16,17,21^ Similarly, van Buchem disease is an allelic HBM condition with less severe pathology that is caused by deletion of a 52 kb homozygous non-coding segment downstream from the *SOST* gene that is essential for *SOST* transcription, but unnecessary for its embryonic transcription.^21–23^

Sclerosteosis is characterised by generalised progressive skeletal overgrowth with thick trabeculae and cortices, dysplastic/absent nails, bony or cutaneous syndactyly and recurrent, acute facial nerve palsy.^24–27^ Facial distortion due to forehead bossing and mandibular overgrowth also arises in early childhood.^24,28^ Early-life conductive hearing loss emerges due to excess bone narrowing of the external auditory canal and reduction of middle ear size.^28–30^ Bone encroaches ossicles and oval/round cochlear windows within the otic capsule and surgical bone removal is often required to facilitate hearing-aid use.^29^ Although bone does not grow into the cochlea, narrowing of the internal auditory canal leads to vestibulocochlear nerve entrapment and sensorineural hearing loss, addressable only by risky canal-widening surgery.^28,29^ Bone-anchored hearing aids, cochlear or brain stem implants are options if conductive hearing aids become ineffective.^29,30^

Cranial nerve entrapment by HBM may also cause neuralgia, anosmia and vision loss.^28,31^ Raised intracranial pressure (RICP), the primary concern for young patients, arises due to restricted intracranial volume, likely coupled with jugular vein compression and jugular canal narrowing.^32,33^ Skull hyperostosis can also engender intracranial hypertension, with brainstem compression, foramen magnum narrowing and sudden death. Furthermore, disruption of cerebrospinal fluid flow around the spinal cord or lower brain stem may precipitate cervical syrinx and interfere with neuronal impulse transmission.^32–35^ To avoid RICP, surgical occipitoparietal/foramen magnum decompression is performed in the first decade of life, when thinner neighbouring bones make surgery easier.^33^ However, decompressive procedures often need to be repeated, yielding unique problems in these patients where laryngeal intubation is challenging due to mandible overgrowth and restricted gape.^33^ These complicated surgical considerations in sclerosteosis patients make development of a pharmacological treatment critical to improving their life quality and expectancy.

Absence of primarily osteocyte-secreted functional sclerostin, which regulates osteoblast, osteocyte and osteoclast function via paracrine control of canonical Wnt/β-catenin, results in excessive canonical Wnt/β-catenin signalling underpinning this HBM phenotype.^36–38^ It is therefore hypothesised that an anti-sclerosteosis drug may fully or partially replicate the normal physiological roles of sclerostin to reverse this excessive Wnt/β-catenin pathway activation. The canonical Wnt-signalling pathway, thoroughly reviewed by Nusse and Clevers, presents many nodes that offer pharmaceutical opportunities for pathway inhibition.^39,40^ Several Wnt/β-catenin signalling inhibitors antagonise Wnt ligands and receptors to reduce Wnt signalling.^41,42^ Alternatively, porcupine (PORCN) inhibitors block Wnt secretion and subsequent Wnt/β-catenin signalling by preventing palmitoylation of Wnt proteins in the endoplasmic reticulum.^40,43–45^ Some agents directly/indirectly affect the β-catenin destruction complex.^40^ For example, degradation of AXIN, the rate-limiting component of the β-catenin destruction complex, can be suppressed by inhibiting tankyrase (TNKS), a negative regulator of the destruction complex.^46,47^ Within the cell nucleus, downstream expression of Wnt target genes can be suppressed by targeting transcription factor 4 (TCF4).^48^ Moreover, some inhibitors suppress β-catenin/TCF-mediated transcription of Wnt target genes by binding competitively to CREB-binding protein (CBP).^49,50^ These different elements of the canonical Wnt-signalling pathway are currently under exploration for potential cancer treatments, with many molecules in various stages of development.^40,51^ Herein, small molecules that inhibit different nodes of the Wnt/β-catenin pathway were investigated in vitro, and the most effective were pursued in vivo by evaluating skull and peripheral bone pathology in a preclinical mouse model of sclerosteosis.

## Results

### LGK974 inhibits osteoblast activity and mineralisation

MC3T3-E1 cells were used for initial candidate screening. Cultures were optimised for osteoblast growth and mineralisation prior to initial screening, which identified that 7 days in osteogenic media (Alpha-MEM, 10% FBS, 1% Ab/Am, 50 µg/mL L-ascorbic acid-2-phosphate (AA2P)), before switching to osteogenic media further supplemented with 2 mmol/L β-glycerophosphate (βGP) for an additional 7 days produced optimal osteoid formation and mineralisation (Fig. S1).

To identify candidate compounds, MC3T3-E1 cells were treated with small molecule inhibitors (LGK974, lorecivivint, ICG001, PNU74654 and XAV939) that target distinct Wnt/β-catenin pathway nodes at the osteoblast differentiation-inducing point (Fig. S2 and Table S1). PNU74654 and XAV939 did not affect cell viability or mineralisation, as assessed by colorimetric and matrix staining assays, respectively. LGK974, lorecivivint and ICG001 displayed toxicity at >1 μmol/L. However, 0.1 μmol/L LGK974 reduced mineralisation to levels similarly observed within the non-osteogenic control group without negatively affecting cell viability. LGK974 was therefore selected to take forward for further in vitro investigation.

**Fig. 1:**
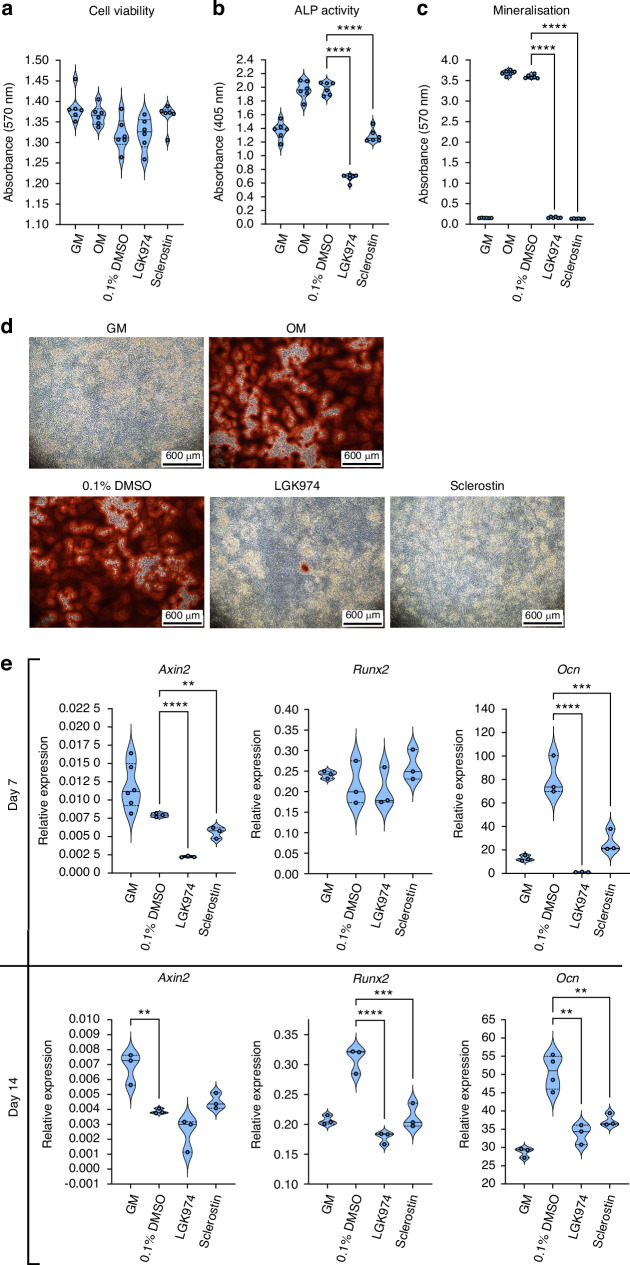
LGK974 inhibits osteoblast activity and mineralisation. Osteoblasts (MC3T3-E1) were treated with 0.1 μmol/L LGK974 and 0.25 μmol/L sclerostin to compare efficacy. DMSO (0.1%) was used as vehicle control. Osteogenic media (OM): growth media (GM) supplemented with 50 μg/mL ascorbate-2-phosphate and 2 mmol/L β-glycerophosphate. **a** Effect of LGK974 on cell viability, after 14 days. **b** LGK974 and sclerostin effect on ALP activity by day 7. **c** Effect of LGK974 and sclerostin on mineralisation after 14 days. **d** Representative light microscope images of MC3T3 cells stained with Alizarin Red S at day 14. **e** Expression of *Axin2* (a Wnt signalling marker), *Runx2*, and *Ocn* (early and late osteoblast markers respectively) on days 7 and 14. Assays were performed using three technical replicates (*n* = 3-6/group). For all graphs, means of each group are indicated by a solid line, and upper and lower dashed lines represent quartiles. Statistical comparisons: **P ≤ 0.01, ***P ≤ 0.001 and ****P ≤ 0.000 1

Comparison of 0.1 μmol/L LGK974 to 0.25 μmol/L sclerostin demonstrated that neither treatment negatively affected MC3T3-osteoblast viability, however both treatments supressed alkaline phosphatase (ALP) activity by day 7 (65.8%; *P* < 0.000 1 and 34.8%; *P* < 0.000 1, respectively) and mineral deposition by day 14 (95.4%; *P* < 0.000 1 and 96.3%; *P* < 0.000 1, respectively), similar to that observed in non-osteogenic control groups (Fig. 1a–d). Furthermore, LGK974 and sclerostin treatment decreased *Axin2* (Wnt signalling marker) expression (71.8%; *P* < 0.000 1 and 30%; *P* < 0.01, respectively) during osteoblast differentiation (day 7), but not in mature osteoblasts (day 14; Fig. 1e). In contrast, *Runx2* (key osteogenic transcription factor) expression was not affected by either treatment on day 7, however expression was reduced (LGK974: 42.4%; *P* < 0.000 1 and sclerostin: 31.4%; *P* < 0.001) to levels identical to non-osteogenic control groups by day 14. Moreover, *Ocn* (osteoblast differentiation marker) expression was reduced at both time points (day 7: 98.6%; *P* < 0.000 1 and 67.1%; *P* < 0.001, LGK974 and sclerostin, respectively; day 14: 33.3%; *P* < 0.01 and 26.2%; *P* < 0.01, LGK974 and sclerostin, respectively), together suggesting that LGK974 functions identically to sclerostin to supress Wnt signalling and subsequent MC3T3-osteoblast differentiation and maturation.

The effects of LGK974 translate to primary osteoblast cultures, whereby 0.1 μmol/L LGK974 ablated primary calvarial osteoblast mineralisation after 21 days of treatment (Fig. S3a–d). To examine whether the observed effects of LGK974 translate to human osteogenic cultures, TERT-immortalised human mesenchymal stromal cells (hMSC) were differentiated towards the osteoblast lineage in the presence of LGK974 and cell viability/ALP activity was assessed. This revealed that high dose (10 μmol/L) LGK974 reduced hMSC viability, with lower concentrations displaying no negative effect on cell viability (Fig. S3e). After 21 days, 5 μmol/L LGK974 reduced hMSC ALP activity (33%, *P* < 0.01), indicating similar LGK974 effectiveness in human osteoblasts (Fig. S3f).

### LGK974 treatment downregulates the Wnt signalling pathway and ossification processes in osteoblasts

**Fig. 2:**
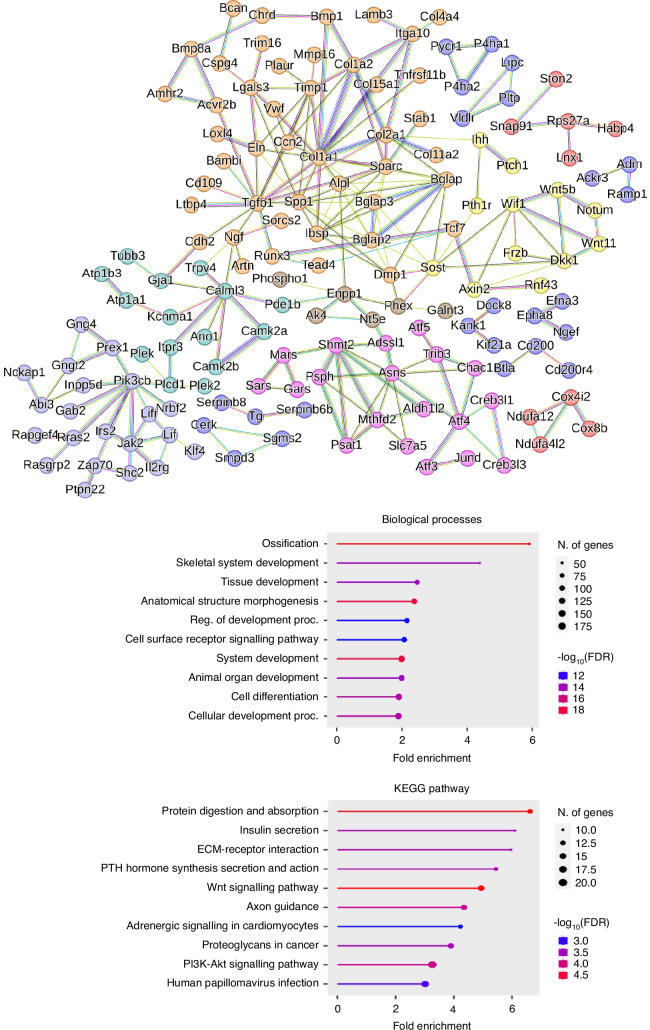
LGK974 treatment downregulates ossification and the Wnt signalling pathway. (Top) Protein-protein interaction network and pathway enrichment analysis from LGK974 treated MC3T3 bulk-RNAseq of day 7 downregulated DEGs (*n* = 3/treatment condition and time point). Note large ossification network (orange) and connected Wnt pathway network (yellow). (Bottom) Biological process and KEGG pathway enrichment analysis of >2-fold downregulated DEGs with LGK974 treatment. Note the 5-fold enrichment of Wnt signalling pathway (KEGG) and 6-fold enrichment in ossification (biological processes) related genes with LGK974 treatment

RNAseq analysis of MC3T3 osteoblast-like cells treated with LGK974 throughout osteogenic differentiation identified the greatest changes in gene expression on day 7 compared to day 14 (Fig. S4), and thus subsequent pathway enrichment analysis was focused on day 7 differentially expressed genes (DEGs). Interaction networks generated via STRING analysis identified a high-confidence, substantially connected system between the downregulated DEGs with LGK974 treatment, with Wnt responsive genes including *Axin2*, *Wnt5b*, *Sost*, *Frzb* (highlighted in yellow) present (Fig. 2). Most notably, ossification was identified as the top downregulated biological process (6-fold enrichment; 49 genes) whilst Wnt signalling was highlighted as the fifth most downregulated KEGG pathway (5-fold enrichment; 16 genes identified) as determined by pathway enrichment analysis. Due to the interconnected nature of *Axin2* in our RNAseq network, and since it is currently used as a biomarker in clinical investigation, we also selected this to show target engagement in our in vivo studies (gene and protein level).^52^ Day 7 and Day 14 DEGs, and correlated DEGs between time points can be found in Supplementary File 2.

### Osteoclast differentiation and activity are unaffected by low LGK974 concentrations

**Fig. 3:**
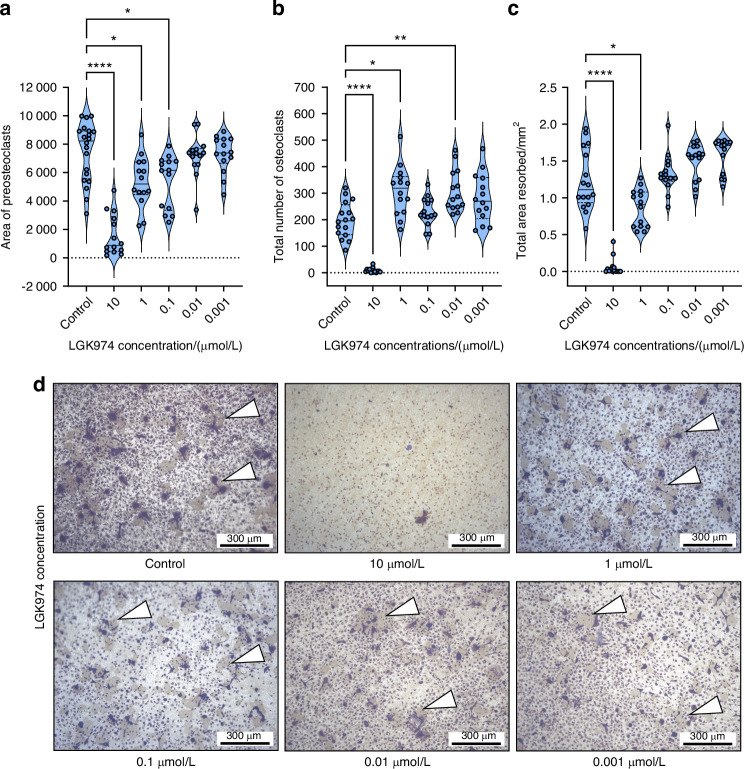
Osteoclast differentiation and activity are unaffected by lower LGK974 concentrations. Quantitative analysis of TRAP-stained primary osteoclasts on dentine discs after 7 days of culture and treatment with LGK974 (0.001 to 10 μmol/L) or other Wnt pathway inhibitors. **a** Area of preosteoclasts and (**b**) total number of TRAP^+^ mature osteoclasts were assessed using the ImageJ Ilastik plugin. **c** Total area resorbed by osteoclasts was calculated using an automated counting method in ImageJ. **d** Representative light microscopy images of osteoclasts treated with different concentrations of LGK974. White arrows indicate resorption pits. For all graphs, means of each group are indicated by the solid line, and upper and lower dashed lines represent quartiles. *n* ≥ 14/group. *P ≤ 0.05, **P ≤ 0.01, and ****P ≤ 0.000 1

Sclerostin does not appear to directly regulate osteoclast differentiation or activity.^38^ Therefore, a therapeutic sclerostin mimetic would ideally also have no direct effect on osteoclasts. At high concentrations (>1 μmol/L), LGK974 reduced preosteoclast area, total osteoclast number and resorbed area (Fig. 3a–d). However, total osteoclast number and resorbed area were unaffected by 0.1 μmol/L LGK974 (effective in osteoblasts). Moreover, little to no effect was observed in preosteoclast area.

These in vitro results show that 0.1 μmol/L LGK974 mimics sclerostin function, suppressing osteoblast mineralisation without affecting osteoblast viability or directly affecting osteoclast viability and activity. LGK974 was thus pursued for its in vivo therapeutic potential.

### LGK974 results in bone morphometric changes in the *Sost*^-/-^ skull

**Fig. 4:**
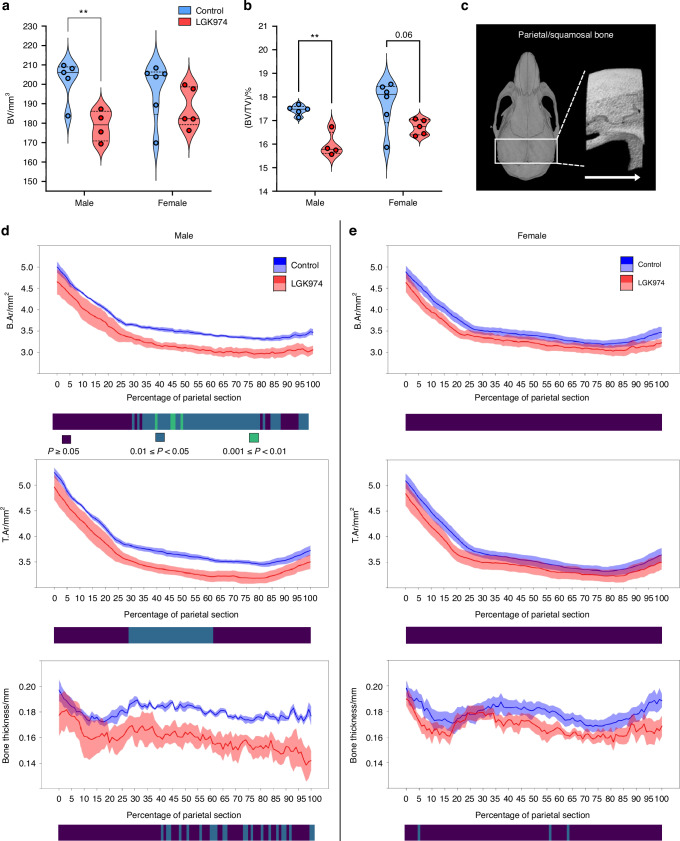
LGK974 treatment decreases bone morphometric parameters in the *Sost*^-/-^ skull. **a** Effect of LGK974 on the cortical bone volume (BV) and (**b**) bone volume fraction (BV/TV) of *Sost*^-/-^ whole skulls (male and female; **P ≤ 0.01). **c** Representative images from a male *Sost*^-/-^ mouse to highlight the analysed region between the anterior lambdoid and coronal sutures (white rectangle). 2D analysis was done from anterior to posterior (white arrow) of the selected region. **d** Bone and tissue area (B.Ar and T.Ar) and thickness of the whole parietal bone section of male treated and untreated *Sost*^-/-^ mice. **e** The same parameters as '**d**', but for females. For all graphs, means of each group are indicated by the solid line, SEM by coloured area. Blue and red represent vehicle and LGK974 treated groups, respectively. Heatmap: dark blue: *P* ≤ 1; green: *P* ≤ 0.05; light blue: *P* ≤ 0.01. Group sizes were *n* = 5 treated females, *n* = 6 non-treated females, *n* = 4 treated males and *n* = 5 non-treated males

Male and female *Sost*^-/-^ mice were treated with 6 mg/kg LGK974 (reported to impair trabecular/cortical bone mass in wild-type mice) for 4 weeks to determine whether PORCN inhibition modifies the HBM phenotype in this mouse sclerosteosis model.^43^ As skull pathologies result in the most severe sclerosteosis patient symptoms, LGK974 treatment on *Sost*^*-/-*^ mouse skulls was initially evaluated. No differences were observed in the foramen magnum diameter and tissue volume (TV) of both male and female LGK974-treated mouse skulls (Table S3). However, a decrease in skull bone volume (BV) and percentage bone volume (BV/TV; 11.6%; *P* < 0.01 and 8.4%; *P* < 0.01, respectively) was observed in male mice (Fig. 4a, b), whilst BV/TV in females showed a near significant downward trend (5.6%; *P* = 0.06). In males, LGK974 also reduced bone area (B.Ar), tissue area (T.Ar) and bone thickness across parietal and squamosal bones (Fig. 4c, d) but no such changes were observed in females (Fig. 4e).

### LGK974 reduces bone morphometry of the middle and inner ear bones

**Fig. 5:**
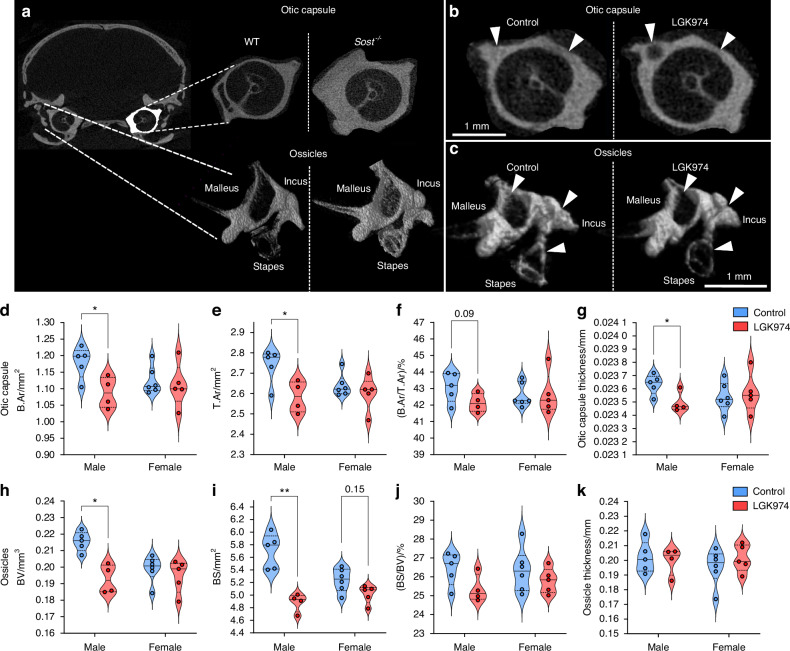
LGK974 treatment reduces bone morphometric parameters in the *Sost*^-/-^ middle and inner ear. **a** Otic capsule region of interest highlighted in white. Representative 2D µCT images of wild type (WT) and *Sost*^-/-^ otic capsules are shown on the top right. Representative 3D µCT images of WT and *Sost*^-/-^ ossicles are shown below the otic capsules. **b** Representative 2D µCT images of the inner ear otic capsules of vehicle control and LGK974 treated mice. **c** Representative 3D µCT images of the middle ear ossicles of vehicle control and LGK974 treated mice. Otic capsule (**d**) B.Ar, (**e**) T.Ar (**f**) B.Ar/T.Ar and (**g**) capsule thickness of male and female *Sost*^-/-^ mice treated with vehicle and LGK974. **h** BV, (**i**) bone surface (BS), (**j**) bone surface fraction (BS/BV) and (**k**) ossicle thickness of male and female treated and untreated *Sost*^-/-^ mice. For all graphs, means of each group are indicated by the solid line, and upper and lower dashed lines represent quartiles. Blue and red represent vehicle and LGK974 treated groups, respectively. *P ≤ 0.05 and **P ≤ 0.01. Group sizes were *n* = 5 treated females, *n* = 6 non-treated females, *n* = 4 treated males and *n* = 5 non-treated males

Sclerosteosis patients suffer from HBM-linked conductive and sensorineural hearing loss.^3^ Interestingly, an HBM phenotype in the ossicles and otic capsule of 1-year old *Sost*^-/-^ mice (versus wild type (WT) C57/BL6 mice) was found (Fig. 5a). We therefore examined whether LGK974 modifies this ossicle (malleus, incus and stapes) and otic capsule phenotype. We found that B.Ar and T.Ar (7.8%, 5.6%, respectively; *P* < 0.05, Fig. 5b, d, e), and bone thickness (0.62%; *P* < 0.05, Fig. 5g) were reduced by LGK974 in male otic capsules, whilst a downward trend (2.2%; *P* = 0.09) was observed in bone percentage (Fig. 5f). Ossicle BV and bone surface (BS) were also significantly reduced by LGK974 in males (8%; *P* < 0.05 and 11.1%; *P* < 0.01, respectively), however, female *Sost*^-/-^ mice were unaffected (Fig. 5c, h, i). LGK974 treatment had no effect on ossicle bone surface percentage (BS/BV) or overall thickness in male and female mice (Fig. 5j, k).

### LGK974 reduces lumbar vertebral bone morphometry and bone turnover marker expression in murine sclerosteosis

**Fig. 6:**
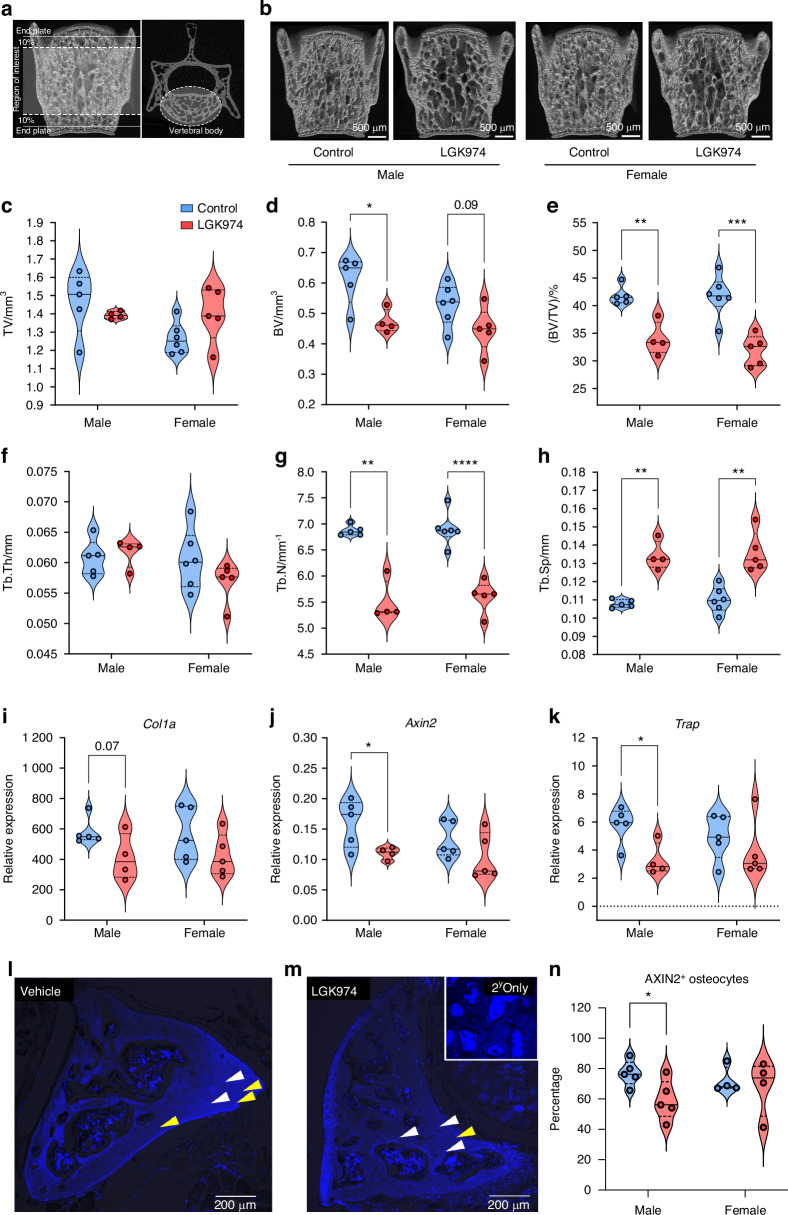
LGK974 treatment results in reduced bone morphometric parameters and bone turnover marker expression in *Sost*^-/-^ lumbar vertebrae. **a** Vertebral regions selected for µCT analysis highlighted in white. **b** Representative 3D µCT images of the L4 vertebral bodies of treated and untreated *Sost*^-/-^ mice (male and female). Effect of LGK974 treatment on L4 lumbar vertebral body (**c**) TV, (**d**) BV, (**e**) BV/TV, (**f**) trabecular thickness (Tb.Th), (**g**) number (Tb.N) and (**h**) space (Tb.Sp). Relative (**i**) *Col1a*, (**j**) *Axin2* and (**k**) *Trap* gene expression in *Sost*^-/-^ lumbar vertebrae. **l**, **m** Representative IHC (AXIN2) images of vehicle and LGK974 treated male *Sost*^-/-^ lumbar vertebrae, with secondary (2^y^) antibody only control included as inset in (**m**). Black = AXIN2 positive osteocytes (yellow arrows) and Blue = AXIN2 negative osteocytes (white arrows). **n** AXIN2 positive (AXIN2^+^) osteocytes (statistical analysis; Welch’s t-test). For all graphs, means of each group are indicated by the solid line, and upper and lower dashed lines represent quartiles. Blue and red represent vehicle controls and LGK974 treated groups, respectively. *P ≤ 0.05, **P ≤ 0.01, ***P ≤ 0.001 and ****P ≤ 0.000 1

LGK974 treatment significantly decreased BV (22.8%, *P* < 0.05) in male *Sost*^-/-^ vertebrae only (Fig. 6c, d; Table S3). For other *Sost*^-/-^ vertebral trabecular bone parameters, LGK974-treated males and females behaved similarly, with trabecular bone volume fraction (BV/TV; 18.8%; *P* ≤ 0.01 and 23.6%; *P* ≤ 0.001, respectively) and trabecular number (Tb.N; 19.9%; *P* < 0.01 and 18.7%; *P* < 0.000 1, respectively) significantly reduced. Trabecular space (Tb.Sp) increased with LGK974 treatment (24.3%; *P* < 0.01 and 23.5%; *P* < 0.01, respectively), whilst trabecular thickness remained unaffected (Fig. 6a, b, e–h).

To underpin the molecular events that result in the distinct bone changes observed with LGK974 treatment in vivo, gene expression analysis was conducted on the vertebrae of male and female vehicle/LGK974 treated *Sost*^-/-^ mice. A trend to decreased vertebral *Col1a* (osteoblast marker) expression was observed in male mice (*P* = 0.07) following LGK974 treatment (Fig. 6i), however this was not apparent in females. However, the relative expression of *Axin2* (Wnt-signalling marker) and *Trap* (osteoclast marker) were significantly reduced (31.2%; *P* < 0.05 and 43.3%; *P* < 0.05, respectively; Fig. 6j-k) in males but not females. This indicates a decrease in bone remodelling events in male LGK974 treated mice, indeed it is known that osteoblast and osteoclast activity is closely coupled.^53^ Interestingly, osteoclasts do not appear to play a major role in the pathological modification of bone in this model as pamidronate treatment, whilst active against isolated osteoclasts, had no effect on bone parameters in vivo (Fig. S9). Notably, AXIN2 protein expression in osteocytes of male mice was significantly decreased (23.1%; *P* < 0.05) by LGK974 treatment, aligning with the *Axin2* gene expression findings (Fig. 6l-n).

### LGK974 reduces tibial bone mass and modifies architecture in murine sclerosteosis

**Fig. 7:**
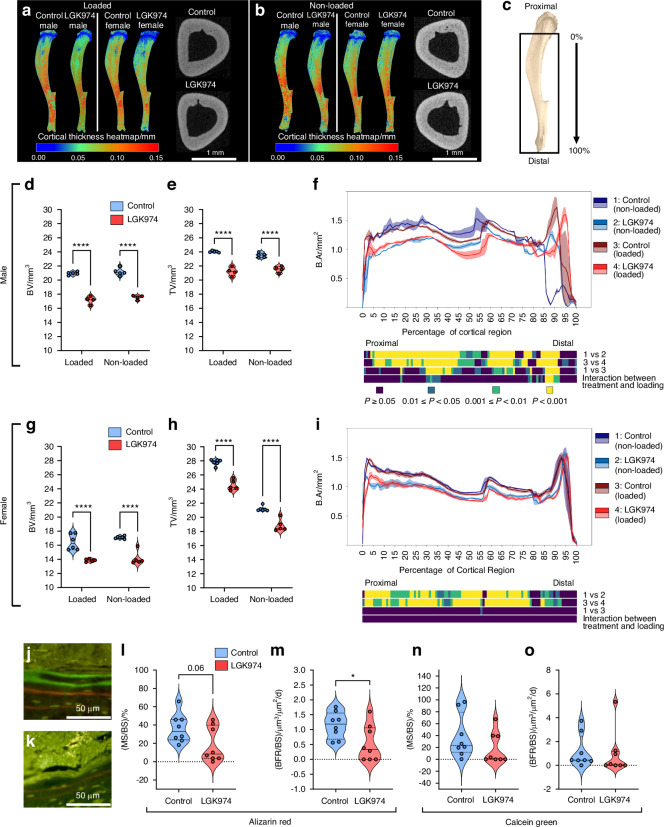
LGK974 treatment reduces tibial cortical bone morphometric parameters in *Sost*^-/-^ mice. **a** Colour coded (heat map of cortical bone thickness), representative 3D µCT images of whole, loaded tibiae (male and female) treated with vehicle and LGK974. Representative 2D µCT cross sections of loaded tibiae are also shown. **b** Cortical thickness heat maps of whole, non-loaded tibiae (male and female) treated with vehicle control and LGK974. Representative 2D µCT cross sections of non-loaded tibiae are also shown. **c** Representative image of a *Sost*^-/-^ mouse tibia that indicates the analysed region (black rectangle). Effect of LGK974 on cortical (**d**, **e**) bone and tissue volume (BV and TV), and (**f**) bone area (B.Ar) of *Sost*^-/-^ male non-loaded and loaded tibiae. **g**–**i** Effect of LGK974 on the same cortical parameters of female tibiae. Representative images of alizarin red (days 4 and 11) and calcein green (days 18 and 25) labelled tibial cortical bone surfaces of male (**j**) control and (**k**) LGK974 treated *Sost*^-/-^ mice. **l** Percentage mineral surface (MS/BS) and (**m**) bone formation rate (BFR/BS) of control and LGK974 treated male loaded tibia (alizarin red dual labelled). (**n**) Percentage mineral surface (MS/BS) and (**o**) bone formation rate (BFR/BS) of control and LGK974 treated male loaded tibia (calcein green dual labelled). Line graphs (**f**, **i**) represent means ± SEM (1 = non-loaded control (dark blue); 2 = non-loaded LGK974 treated (light blue); 3 = loaded control (maroon); 4 = loaded LGK974 treated (red)). Graphical heat maps below line graphs summarise statistical differences of the above groups 1-4 at specific matched locations along the analysed length of the tibiae and represent the overall effect of LGK974 treatment (dark blue *P* ≥ 0.05, blue 0.01 ≤ *P* < 0.05, green 0.001 ≤ *P* < 0.01, yellow *P* < 0.001). Interaction is between LGK974 treatment and loading. For violin plots, means of each group are indicated by the solid line, and upper and lower dashed lines represent quartiles. Blue and red represent vehicle controls and LGK974 treated groups, respectively. *P ≤ 0.05, **P ≤ 0.01 and ****P ≤ 0.000 1. Group sizes were *n* = 5 treated females, *n* = 6 control females, *n* = 4 treated males and *n* = 5 co*n*trol males. Dynamic histomorphometry group sizes were *n* = 4 control and *n* = 4 treated males, with two levels analysed for each tibia

To explore if LGK974 sensitivity extends to long bones and whether this response interacts with localised biomechanical cues, we also examined loaded and non-loaded tibiae in control and LGK974-treated *Sost*^-/-^ mice. Despite a lack of significant effects on body weight (Fig. S5), we found that comparison across non-loaded (left) and loaded (right) tibiae revealed significant reductions in cortical mass in LGK974 treated male and female *Sost*^-/-^ mice, which extended along extensive portions of the bone length in both males and females (Fig. 7a-i). Male BV and TV were decreased by LGK974 treatment in both loaded (BV: 18.1%; *P* < 0.000 1; TV: 11.7%; *P* < 0.01) and non-loaded (BV: 17.2%; *P* < 0.000 1; TV: 8.46; *P* < 0.001) tibiae (Fig. 7d, e). Likewise, BV and TV were also reduced in non-loaded (BV: 17.3%; *P* < 0.000 1; TV: 11.1%; *P* < 0.01) and loaded (BV: 15.7%; *P* < 0.000 1; TV: 11.7%; *P* < 0.001) female tibiae (Fig. 7g, h). Notably, in females, TV was markedly higher in loaded than non-loaded limbs. Load application (left/right comparison) in untreated *Sost*^-/-^ mice provoked more marked tibial architectural changes in males than in females (Fig. 7f and i). Intriguingly, a clear interaction between LGK974 treatment and localised load-induced modulation of cortical mass extended to male *Sost*^-/-^ mice only (B.Ar/T.Ar; Fig. S6b); most markedly in more proximal tibial regions but also distally. Interestingly, no such load:LGK974 interaction was evident in female *Sost*^-/-^ mice, where loading dramatically increased T.Ar in the proximal tibia to contribute to overall and localised diminution in B.Ar/T.Ar in both control and LGK974-treated mice (Fig. S6c, d). Furthermore, LGK974 treatment reduced bone formation rate (BFR/BS; 56.5%; *P* < 0.05), with a trend towards a reduction in bone mineral surface (MS/BS; 53%; *P* = 0.06) in the cortical compartment in male tibiae during the early loading period (alizarin red labelled), whilst no effect was seen during the non-loading phase (calcein labelled; Fig. 7j–o). Conversely, mineral apposition rate (MAR) remained unaffected by LGK974 (Fig. S6e, f).

These data indicate that the bone-modifying LGK974 effects in *Sost*^-/-^ mice are also seen in long bones and that interaction with mechanical load-related regulation of bone mass is evident only in males.

**Fig. 8:**
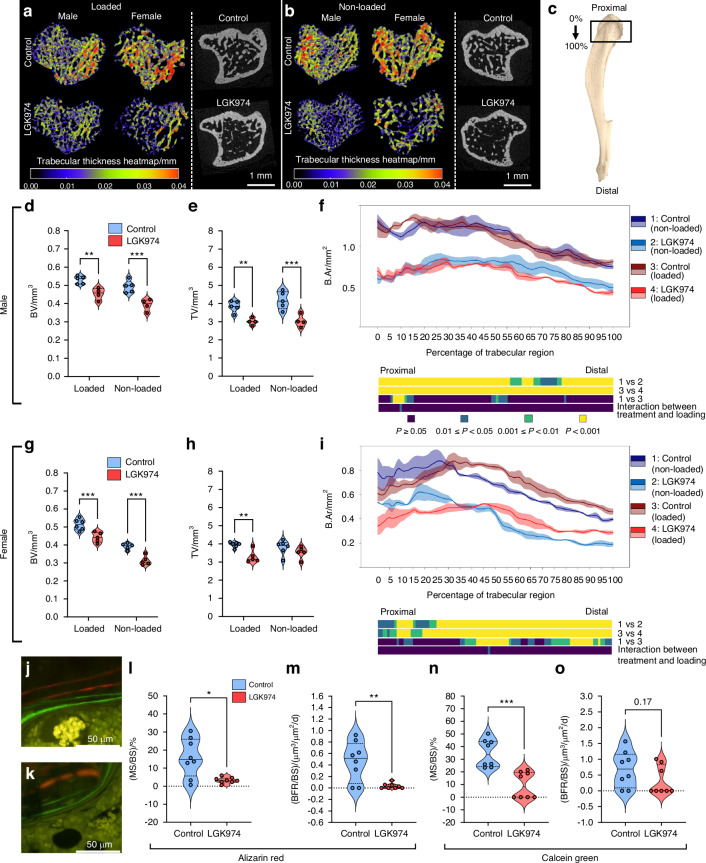
LGK974 treatment reduces tibial trabecular bone morphometric parameters in *Sost*^-/-^ mice. **a** Colour coded (heat map of trabecular bone thickness), representative 3D µCT images of the proximal region of loaded male and female tibiae (male and female) treated with vehicle and LGK974. Representative 2D µCT cross sections of the proximal region of loaded tibiae are also shown. **b** Trabecular thickness heat maps of the proximal end of non-loaded tibiae (male and female) treated with vehicle control and LGK974. Representative 2D µCT cross sections of non-loaded tibiae are also shown. **c** Representative image of a *Sost*^-/-^ mouse tibia, with a black rectangle indicating the analysed region. Effect of LGK974 on trabecular (**d**, **e**) bone and tissue volume (BV and TV), and (**f**) bone area (B.Ar) of *Sost*^-/-^ male non-loaded and loaded tibiae. **g**–**i** Effect of LGK974 on the same cortical parameters of female tibiae. Representative images of alizarin red (days 4 and 11) and calcein green (days 18 and 25) labelled tibial cortical bone surfaces of male (**j**) control and (**k**) LGK974 treated *Sost*^-/-^ mice. **l** Percentage mineral surface (MS/BS) and (**m**) bone formation rate (BFR/BS) of control and LGK974 treated male loaded tibia (alizarin red dual labelled). **n** Percentage mineral surface (MS/BS) and (**o**) bone formation rate (BFR/BS) of control and LGK974 treated male loaded tibia (calcein green dual labelled). Line graphs (**f**, **i**) represent means ± SEM (1 = non-loaded control (dark blue); 2 = non-loaded LGK974 treated (light blue); 3 = loaded control (maroon); 4 = loaded LGK974 treated (red)). Graphical heat maps below line graphs summarise statistical differences of the above groups 1-4 at specific matched locations along the length of the analysed region and represent the overall effect of LGK974 treatment (dark blue *P* ≥ 0.05, blue 0.01 ≤ *P* < 0.05, green 0.001 ≤ *P* < 0.01, yellow *P* < 0.001). Interaction is between LGK974 treatment and loading. For violin plots, means of each group are indicated by the solid line, and upper and lower dashed lines represent quartiles. Blue and red represent vehicle controls and LGK974 treated groups, respectively. *P ≤ 0.05, **P ≤ 0.01 and ****P ≤ 0.000 1. Group sizes were *n* = 5 treated females, *n* = 6 control females, *n* = 4 treated males and *n* = 5 co*n*trol males. Dynamic histomorphometry group sizes were *n* = 4 control and *n* = 4 treated males, with two levels analysed for each tibia

Comparison across metaphyseal regions in loaded (right) and non-loaded (left) tibiae revealed reductions in trabecular bone mass and microarchitecture in male and female LGK974 treated *Sost*^-/-^ mice (Fig. 8a–i and Fig. S7). LGK974 produced significant decreases in trabecular BV, TV and B.Ar in male and female *Sost*^-/-^ mice (Fig. 8d–i; Table S3). In loaded tibiae, LGK974 treatment decreased BV by 14.5% and 13.7% in male and female mice, respectively (*P* < 0.01), whilst reductions in TV were 21.8% (*P* < 0.001) for males and 16.6% (*P* < 0.01) for females (Fig. 8d, e, g and h). BV was also decreased in male and female non-loaded tibiae (20.4 and 21.7%; male and female, respectively; *P* < 0.001). Notably, there was no change in non-loaded TV in females in response to LGK974 treatment, but male TV was reduced by 27.2% (*P* < 0.01) (Fig. 8e, h). In females only, loading enhanced B.Ar in mid-distal trabecular regions in untreated *Sost*^-/-^ mice, but load:LGK974 interaction was not evident in either males or females (Fig. 8f, i; Fig. S7a–d). LGK974 also significantly reduced trabecular thickness (Tb.Th) and number (Tb.N), whilst increasing spacing (Tb.Sp) in both sexes (Fig. S8). Interestingly, loading of female *Sost*^-/-^ tibiae exposed some regional variation, with decreased Tb.Th proximally and increased distally. During the loading period, LGK974 treatment reduced trabecular MS/BS (76%; *P* < 0.05) and BFR/BS (94%; *P* < 0.01), with MAR indicating a trend to reduction (64%; *P* = 0.055 in male *Sost*^-/-^ mice (Fig. 8j–m; Fig. S7e). MS/BS was also significantly reduced (72.6%; *P* < 0.001) during the latter, non-loading phase of LGK974 treatment, whilst BFR/BS and MAR remained unchanged (Fig. 8n, o; Fig. S7f). Interestingly, the presence of all 4 labels also suggests that there is no aberrant osteoclast-mediated resorption of bone in response to LGK974.

## Discussion

We have found that LGK974, an inhibitor of PORCN-targeted Wnt/β-catenin signalling, limits in vitro osteogenic osteoblast behaviour, akin to sclerostin, and restricts the emergence of several skeletal HBM pathologies that arise in *Sost*^-/-^ mice, which are characteristic of human sclerosteosis.

Critically, we show that LGK974 limits capsule and ossicle otosclerosis, and lowers skull bone mass in *Sost*^-/-^mice, providing evidence of its translational protection against debilitating hearing loss and life-threatening increases in intracranial pressure seen in sclerosteosis patients.

Our prior investigations into use of various forms of recombinant sclerostin, including fusion proteins as a possible sclerosteosis treatment, found that these only partially and modestly corrected the HBM phenotype in the *Sost*^-/-^ vertebra.^54^ This lack of efficacy may be due to low drug exposure, protein stability, protein immunogenicity (anti-drug antibodies were observed in treated animals) or a combination of all, suggesting that recombinant sclerostin would unlikely be an effective long-term treatment for sclerosteosis.

Exploration of alternative approaches to suppress HBM in sclerosteosis patients led us to evaluate small molecule inhibitors that target different Wnt/β-catenin signalling pathway nodes.^40^ Although most of these screened candidates were either cytotoxic or failed to appropriately modify in vitro osteoblast behaviour (Fig. S2), we found that LGK974 showed greatest promise. PORCN inhibition by LGK974 blocks palmitoylation and exit of Wnt from the endoplasmic reticulum, thereby inhibiting its signalling without affecting normal Wnt-dependent tissue function. Indeed, downregulated Wnt signalling and ossification seen in our in vitro osteoblast cultures via RNAseq analysis support the notion that LGK974 functions to suppress Wnt driven osteogenic processes via porcupine inhibition. Promise in targeting PORCN has previously been substantiated in studies where LGK974 treatment reduced bone mass in wild type mice and humans.^43,44^

Our data in mouse and human osteoblasts found that ALP activity, mineralisation and Wnt osteoblast marker expression were all reduced by in vitro LGK974 exposure, displaying similar effects to exogenous sclerostin.^55,56^ The mimicking of sclerostin’s properties is further supported by our data showing that low LGK974 concentrations (≤0.1 μmol/L), akin to sclerostin, did not directly affect osteoclast number or function.^37,38^ As PORCN inhibitor-mediated bone loss has already been reported in wild type mice and humans, we explored if LGK974 similarly modified the HBM phenotype known to exist in *Sost*^-/-^ mice, a well-studied sclerosteosis model.^43,44,57^ Skull sclerosis and hyperostosis are prominent and severe pathologies in sclerosteosis patients, which prompted us to measure a range of skull bone parameters in the *Sost*^-/-^ mice.^24,33^ This revealed LGK974-mediated decreases in cranial bone volume and parietal bone thickness, whilst foramen magnum diameter was not significantly affected, showing a trend for a reduction in only male *Sost*^-/-^ mice. Whilst higher or extended dosing may lead to more marked changes, it is apparent that an exploration of the natural history of these phenotypes in both sexes of *Sost*^-/-^ mice (currently ongoing) will further our appreciation of the extent to which they reflect age-related onset in the human patient populations.

Conductive/sensorineural hearing loss caused by HBM ear bone changes is a prominent phenotype in sclerosteosis patients.^29,30^ Our studies revealed that the scale of the sclerosis in *Sost*^-/-^ males was compellingly limited by LGK974 treatment. This indicates that long-term LGK974 treatment may prevent or minimise the severe hearing loss developing in human patients.

We found that LGK974 treatment reduced trabecular bone mass in the lumbar vertebrae more robustly than our previously investigated recombinant sclerostin constructs.^54^ Treatment also significantly decreased both cortical and trabecular bone mass in the tibiae of *Sost*^-/-^ mice, both basally and in loaded limbs. Decreased Wnt activity, indicated by decreased *Axin2* expression, likely drives the reduction in bone by reducing osteoblast function and activity, as evidenced by the decreased mineral surface and bone formation rate. Due to osteoblast-osteoclast coupling, this reduced osteoblast activity may contribute to a decrease in osteoclast activity, as LGK974 treatment also reduced vertebral *Trap* expression. However, bisphosphonate-mediated blocking of osteoclasts in *Sost*^-/-^ mice did not influence tibial bone parameters (Fig. S9), suggesting that osteoclasts have a limited role in pathological bone formation in *Sost*^-/-^ mice. These data suggest that osteoblast-mediated bone formation in *Sost*^-/-^ mice is the major driver of bone pathology, and that changes in osteoclast markers following LGK974 treatment is solely due to osteoblast-osteoclast coupling. Differences in the cortical bone resulting from LGK974 treatment were more marked in non-loaded than loaded male tibiae, indicating that a significant interaction occurs between the response to mechanical loading and LGK974-mediated inhibition of PORCN-targeted Wnt/β-catenin signalling in the cortical bone compartment, which is lacking in female *Sost*^-/-^ mice. This lack of load:LGK974 interaction extended to load-induced increases in trabecular mass seen in female *Sost*^-/-^ mice, in which load application did not influence LGK974 efficacy.

As the loading experiments were conducted in young, growing *Sost*^-/-^ mice that likely mirror mechanical load-bearing events experienced in early childhood, a period of heightened load activity, studies in patients should carefully consider the age of the individuals being treated with LGK974.^58–60^ This differing disease severity and LGK974-mediated protection against HBM phenotypes seen in male and female *Sost*^-/-^ mice contributes to growing evidence showing the relevance of sexually dimorphic mechanisms that mediate bone homoeostasis and the onset of various disease, with potential links to how bone-modifying drugs exert their efficacy.^61^

The impact of our findings extends to the potential cohorts that should be chosen for treatment with LGK974. Thus, it is interesting that sclerosteosis heterozygous ‘carriers’ also have somewhat higher bone density/Z-score and lower sclerostin levels than normal cohorts, yet they exhibit none of the HBM-linked pathologies.^3,34^ This suggests that defined bone density/Z-score levels could be set as a ‘treat-to-target’ threshold in any LGK974 clinical trials, where the aim would be to effectively convert a homozygous sclerosteosis phenotype, which is linked with pathological HBM, to heterozygous carrier levels lacking overt pathological skeletal changes.

We appreciate that this study has inherent limitations. For instance, LGK974 specificity and effectiveness could be further validated in vitro by treating cells with an alternative PORCN inhibitor and monitoring Wnt pathway activity. It is also possible to enhance our in vivo data by using an extended LGK974 dosing regimen to explore whether this might be more effective in reducing the *Sost*^-/-^ mouse HBM phenotype, especially in females. Furthermore, modifications in dose level may likewise enrich our appreciation of the potential sexually dimorphic responses to LGK974 treatment and its interaction with mechanoadaptive changes in bone mass and architecture by allowing us to determine whether female *Sost*^-/-^ mice require different LGK974 treatment regimens to attain levels of protection against HBM seen in male skulls. In vivo measures of osteocyte, osteoblast and osteoclast activity and function in both *Sost*^-/-^ and wild type mouse bone would serve to further demonstrate LGK974’s mechanism of action, and the monitoring of hearing loss and intracranial pressures could help to expose the extent to which LGK974-related PORCN inhibition influences the emergence of these pathologies. Moreover, only assessing bone formation rate in males, despite convincingly demonstrating that LGK974 has its effect on osteoblasts, restricts our scope to define the limited change in bone formation as a contributor to possible sexual dimorphism. However, analysis of vertebral gene expression and AXIN2 osteocyte positivity appears to suggest that the same reduction in osteoblast-lineage-mediated processes is not observed in female mice in response to LGK974 when compared to males. Monitoring of these pathologies is also potentially non-optimal as there are currently no established guidelines for reporting mouse skull bone phenotypes, and hence may benefit from our continued methodological refinement. Lastly, Wnt signalling is known to be involved in normal tissue homoeostasis and is implicated in liver, lung, skin, cardiovascular and neurodegenerative disease.^45,62^ Thus, targeting Wnt mediators has the potential for adverse effects and modulation of this pathway should be carefully considered. Indeed, exposure-related dysgeusia has been reported in PORCN-inhibitor clinical trials.^52,63^ Moreover, PORCN inhibitors induce intestinal toxicity at very high concentrations, but are well tolerated at efficacious doses.^45,64,65^ Mouse soft tissues (such as lungs, liver, intestines and kidneys) should therefore be collected to study potential extra-skeletal LGK974 effects and further validate the translational potential of LGK974 or other PORCN inhibitors.

It is noteworthy that coincidentally a concurrent study also evaluated PORCN inhibition as an option for treating sclerosing HBM symptoms.^66^ The study by Diegel et al. evaluated if 5/6 weeks of PORCN inhibitor treatment (LGK974 or GNF-6231, another small-molecule) modified long bone architecture and mass in HBM mouse models of aberrant Wnt signalling. They found that PORCN inhibitor treatment dose-dependently decreased femoral and tibial bone mineral content and density, trabecular BMD, and cross-sectional cortical thickness in two strains with *Lrp5* point mutations and in *Sost*^-/-^ mice as measured by peripheral quantitative computed tomography (pQCT). In contrast to our own studies, Diegel et al. commenced treatment in 3-month-old mice and found that these indices of bone architecture were also diminished in wild-type mice. Our work expands upon this to show that the benefits of LGK974 treatment extend to 6-week-old mice, thus increasing the likely translational relevance of our findings to young, immature growing sclerosteosis patients. Unlike Diegel et al., we also assessed skull bone parameters as these are the primary healthcare concerns for sclerosteosis patients. Without our work, it would be impossible to securely extrapolate the LGK974 treatment benefits to the flat, intramembranous skull bones where the major debilitating symptoms emerge in sclerosteosis patients. Likewise, as the studies of Diegel et al. assessed the effect of PORCN inhibitors in female mice only, this would have meant that the possible sexually dimorphic potency of PORCN-targeted Wnt/β-catenin signalling would have remained unknown.^66^

Our study, by comparison, took a highly translational approach by evaluating the effects of LGK974 in younger mice and included males and females.

We identified, for the first time, a potential sexual-dimorphic response to LGK974 treatment in the HBM *Sost*^-/-^ skull, with significant treatment-induced bone decreases evident in calvaria and ears of males only. Interestingly, this could also be due to basal sex differences in *Sost*^-/-^ mice, with studies showing somewhat greater, albeit not significant, bone mass gains in females than males following *Sost* deletion.^57^ These data suggest that care should be taken when planning LGK974 dosing regimens and clinical trials for male versus female patients.

We report that PORCN inhibitor treatment reduces bone mass in various skeletal sites that are known to be profoundly affected by sclerosteosis i.e. skull, ear as well as the spine and limbs. This is the first evidence of a therapeutic approach that effectively limits bone accrual at skeletal sites in mice that are severely affected in sclerosteosis patients, supporting the use of this approach to alleviate the symptoms of sclerosteosis and potentially of other *Wnt*-related HBM conditions, such as Van Buchem disease. The significance of these data is highlighted when the difficulty and danger of the surgical methods currently used to manage the disease are considered. Therapeutic translation would indeed greatly improve the life expectancy and quality of life of these patients and affected families, especially in countries with inadequate public health systems. This, however, requires further investigation of PORCN inhibitor safety and usability in children and adults, and despite the great promise shown herein by LGK974, screening should continue to seek other candidate drugs. It is notable that prior research exploring the basis of HBM in these sclerosteosis patients generated huge advances which led to the development of romosozumab, an FDA-approved osteoporosis therapeutic.^17,67^ It would thus be fitting that sclerosteosis patients should also now benefit from the understanding of bone biology which their condition has helped to acquire. Significant progress towards a sclerosteosis treatment has now been made, however, all collaborators across academia and the pharmaceutical industry continue to have an ethical obligation to progress this research until a successful treatment is identified.

## Materials and methods

### Study design

To identify compounds that could alleviate HBM in sclerosteosis, Wnt pathway inhibitor candidates were screened for their capacity to inhibit activity and mineralisation of osteoblast-like cells. Based on activity and function in different osteoblast (viability, ALP activity, mineralisation, gene expression) and osteoclast models (preosteoclast area, osteoclast number, resorbed area), the most promising drug (LGK974, PORCN inhibitor) was further assessed. Once in vitro efficacy was confirmed, LGK974 was administered to *Sost*^-/-^ mice, a sclerosteosis model. Control and treatment groups consisted of 6-week-old male and female mice (total *n* = 20). Right tibiae were loaded to determine whether LGK974 interacts with mechanoadaptive responses. Mouse skull, ear bones, vertebrae and left (non-loaded) and right (loaded) tibiae were analysed by microcomputed tomography (µCT). All procedures were approved by RVC ethics board and performed according to ARRIVE guidelines, in fulfilment of the UK Animals (Scientific Procedures) Act 1986.^68^

### Wnt pathway inhibitors

Small molecule inhibitors screened in this study (Table S1) were: XAV939 (targets Tankyrase)^46,69,70^; PNU74654 (targets TCF4);^48,71^ ICG001 (targets CREB-binding protein);^49,50^ lorecivivint (indirectly targets GSK3β)^72^ and LGK974 (targets PORCN).^43,44^

### Cell culture

MC3T3-E1 Subclone 14 mouse pre-osteoblasts (ATCC CRL-2594; passage 29) were expanded in complete growth medium (GM; minimum essential medium alpha (alpha MEM) containing 2 mmol/L L-glutamine, ribonucleosides, deoxyribonucleosides, 1% antibiotic-antimycotic (Thermo Fischer Scientific (Waltham, Massachusetts, USA)) and 10% foetal bovine serum (FBS; Sigma-Aldrich, St. Louis, Missouri, USA)) and plated in 96/24 well plates (10^4^ cells/cm^2^) and maintained at 37 °C/5% CO_2_. After 48 h, medium was changed to optimised osteogenic growth medium (Fig. S1; OM: GM supplemented with 50 μg/mL ascorbate-2-phosphate (AA2P) (added from day 0–14) and 2 mmol/L β-glycerophosphate (added from day 7–14)) to promote osteogenic differentiation. On day 0, cells were treated with either 0.1% DMSO (control), <10 μmol/L LGK974 (APExBIO, Houston, Texas, USA), other Wnt inhibitors, or 0.25 μmol/L recombinant mouse sclerostin (gift, UCB Pharma). Treatment was repeated 3x/week and cells were fixed with 4% paraformaldehyde (PFA) after 14 days.

Primary mouse calvarial osteoblasts were isolated and cultured as described.^73^ Calvaria were collected from neonatal C57/BL-6 pups (2–5 days old), washed with 1x DPBS and incubated in 0.25% trypsin for 10 min at 37 °C, after which trypsin solution was discarded. Calvaria were washed with GM, then incubated in 0.2% (w/v) collagenase (Type II collagenase, Clostridium histolyticum) in Hank’s balanced salt solution for 30 min at 37 °C. Collagenase digest was removed and replaced with fresh collagenase solution for 60 min at 37 °C. The final digest and GM from a final wash were collected, centrifuged (1 500 x *g*, 5 min), supernatant discarded, cells resuspended in GM and cultured at 37 °C/5% CO_2_ until confluent. Cells were then plated in 6-well plates (10^5^ cells/well) and maintained at 37 °C/5% CO_2_. From day 0, experimental cells were cultured in OM (with 5 mmol/L β-glycerophosphate) and treated 3x/week in the presence or absence of 0.1 μmol/L LGK974. Cells were fixed with 4% PFA after 21 days.

Immortalised human mesenchymal stromal cells (hMSCs; ATCC SCRC-4000; passage 10) expanded in Dulbecco’s Modified Eagle Medium (DMEM) with glutamax (Thermo Fisher Scientific), 10% FBS and 1% Antibiotic-Antimycotic) were plated in 96/24 well plates (10^4^ cells/cm^2^) and maintained at 37 °C/5% CO_2_ for 48 h. Cells were cultured in OM (expansion medium with 50 μg/mL ascorbate-2-phosphate, 5 mmol/L β-glycerophosphate and 100 nmol/L dexamethasone (Sigma-Aldrich)) and treated with up to 10 μmol/L LGK974 every 2-3 days before being fixed with 4% PFA after 21 days.

Primary murine osteoclasts were prepared as described previously.^74^ Briefly, bone marrow was flushed using DPBS from 6-week-old C57/BL-6 mouse long bones, cultured for 24 h in the presence of 50 ng/mL macrophage colony-stimulating factor (M-CSF) (Peprotech, Cranbury, New Jersey, USA) and 100 nmol/L prostaglandin E_2_ (PGE_2_) (Sigma-Aldrich) in MEM containing 10% FBS, 2 mmol/L L-glutamine, 100 U/mL penicillin, 100 μg/mL streptomycin and 0.25 μg/mL amphotericin. After 24 h cells were seeded onto dentine discs (1×10^6^ cells/5 mm disc) and allowed to adhere for 24 h in MEM containing 200 ng/mL M-CSF, 5 ng/mL RANK-L (Peprotech) and 100 nmol/L PGE_2_. Discs were then transferred to 6-well plates (4 discs/well) and maintained in varying concentrations of LGK974 for 3 days. On day 5, 12 mol/L HCl was added to adjust the medium pH to 7.0, facilitating the initiation of resorption. Treatment with/without LGK974 was continued to day 7 and cells then fixed in 2.5% glutaraldehyde.

### Osteoblast viability, ALP activity and mineralisation assays

Osteoblast viability was assessed on days 7 and 14 using a resazurin assay, which does not require fixation, allowing for downstream analysis.^75^ Briefly, GM was replaced with complete osteoblast growth medium containing 0.2 mmol/L resazurin (APExBIO). Cells were incubated at 37 °C/5% CO_2_ for 1 h and absorbance measured at 570 nm (600 nm reference wavelength, Spark® Cyto plate reader (Tecan, Männedorf, Switzerland)). Alkaline phosphatase (ALP) activity was assessed using *p*-nitrophenyl phosphate (pNPP) substrate.^76^ Cells were washed with prewarmed DPBS after the cell viability assay, followed by the addition of prewarmed pNPP (Sigma-Aldrich) and incubation at 37 °C for 15 minutes. The supernatant was transferred to 96-well non-tissue culture plates and absorbance measured at 405 nm. The calcium content of mineralised matrix was measured on day 14 by staining formalin-fixed cells with 40 mmol/L Alizarin Red S (ARS; pH 4; Sigma Aldrich) and plates scanned using a flatbed scanner (Canon). For quantitation, ARS was dissolved with 10% (w/v) cetylpyridinium chloride (CPC; Sigma-Aldrich) and absorbance was measured at 570 nm.

### Bulk RNA-seq of LGK974 treated MC3T3-osteoblast like cells

MC3T3-E1 Subclone 14 mouse pre-osteoblasts (ATCC CRL-2594; passage 29) were seeded in 24-well cell culture plates (19 000 cells/well) and differentiated in the presence of 0.1 μmol/L LGK974 or DMSO control for 7 or 14 days before RNA extraction (*n* = 3 per treatment condition). Total RNA quantification and integrity were determined via Bioanalyzer 2100 (Agilent) and RNA samples with an integrity number above 9.0 were processed for RNA sequencing at NovoGene (Cambridge, UK). The RNA library was formed by polyA capture using poly-T oligo-attached magnetic beads and subsequently reverse transcribed for cDNA synthesis and library construction. The library was then sequenced using Illumina PE150 technology and clean reads with high-quality data were taken forward for downstream analysis. Paired-end clean reads were aligned to the *Mus Musculus* reference genome and read numbers mapped to each gene. Fragments Per Kilobase of transcript sequence per Millions base pairs sequenced (FPKM) of each gene were calculated based on the length of the gene and reads count mapped to the gene. Differential expression analysis of LGK974 treatment versus DMSO control was performed for both day 7 and 14 time points. Genes with an adjusted *P*-value ≤ 0.05 and a fold change ≥ 2 were considered differentially expressed (DEGs). Pathway enrichment analysis was carried out using ShinyGO 0.80 (sdstate.edu) with ≥2-fold DEGs and a false discover rate (FDR) cut-off set to 0.05 for both upregulated/ downregulated genes individually and input into the STRING: functional protein association networks (www.string-db.org) to determine high confidence protein-protein interactions.

### Gene expression

Gene expression was assessed by qPCR. Briefly, RNA was extracted from cells using ReliaPrep™ RNA Miniprep System (Promega, Madison, Wisconsin, USA). For *Sost*^-/-^ mouse vertebra RNA extraction, samples were suspended in QIAzol (QIAGEN, Hilden, Germany) and homogenised using a Polytron™ probe (Kinematica, Lucerne, Switzerland), followed by RNA extraction using the Zymo Direct-zol™ RNA Miniprep kit (Zymo Research, Irvine, California, USA). cDNA was synthesised (Applied Biosystems™ High-Capacity cDNA Reverse Transcription Kit, Thermo Fischer Scientific) and qPCR analysis was carried out using a qPCRBIO SyGreen Blue Mix (PCR Biosystems) and a RT-PCR CFX connect (BioRad, Hercules, California, USA). Primers are listed in Table S2.

### In vivo study design

Male and female *Sost*^-/-^ mice (6-week-old) were housed in polypropylene cages with environmental enrichment and a 12 h light/dark cycle at 21 ± 2 °C. Bone mass gains in *Sost*^-/-^ are rapid at 4-6 weeks and therefore this age was selected.^77^ Mice had access to RM1 standard diet (LBS Biotechnology, Horley, UK) and water *ad libitum*. Each mouse was weighed prior to dosing. Mice (*n* = 11 animals in vehicle group, 5 males and 6 females; *n* = 9 in LGK974 treatment group, 4 males and 5 females) were subjected to the loading regimen (below), with 2x/week dosing via oral gavage of either vehicle (DMSO; Thermo Fisher Scientific) or 6 mg/kg LGK974 (APExBIO) for the first 2 weeks.^43^ LGK974 was dissolved in DMSO and diluted in a 5% DMSO, 1% carboxymethylcellulose (Sigma-Aldrich) and 0.2% Tween 80 (Sigma-Aldrich) solution. This was followed by 2 weeks of daily dosing (5 days on, 2 days off). Intra-peritoneal (IP) injections of 50 mg/kg alizarin red on days 4/11 and 10 mg/kg calcein on days 18/25 were administered for dynamic histomorphometry. Animals were culled by CO_2_, confirmed by cardiac puncture.

### In vivo loading

*Sost*^-/-^ tibiae have been mechanically loaded previously.^78,79^ Herein, tibiae were loaded in pilot studies with peak 25 N to determine optimal non-bone-damaging load. The maximal load was determined as 21 N and 24.5 N for female and male *Sost*^-/-^ mice, respectively, and peak 20 N loads were deemed optimal for in vivo application. The right knee of 6-week-old *Sost*^*-/-*^ mice was loaded 3x/week under isoflurane anaesthesia for 2 weeks using established methods: 40 cycles (0.05, 0.025 and 9.9 seconds for peak (20 N), rise/fall, and hold (2 N) time, respectively for each episode) with left tibia as contralateral control.^80^

### Microcomputed tomography (µCT) of axial and appendicular skeletal bones

#### Sample preparations, acquisition settings, and morphometrical analysis

Samples were PFA fixed for 48 h, washed with DPBS and stored in 70% ethanol. Right and left hind limbs, L4 and L5 lumbar vertebrae and skulls were wrapped in non-PVC plastic film and scanned using the Skyscan 1172 F (Bruker, Kontich, Belgium). X-ray projection images, taken with a 0.6° rotation step over 180° sample rotation, were acquired (software version 1.5). For limbs and vertebrae, the X-ray tube was operated at 50 kV, 200 μA, 960 ms exposure time using a 0.5 mm aluminium filter and 5 μm voxel size. For skulls, the X-ray tube was operated at 80 kV, 124 μA, 1 900 ms exposure time using a 0.5 mm aluminium filter and 13.5 μm voxel size. Projection images were reconstructed (NRecon version 1.7.4.6, Bruker) with the following settings: *tibiae* **-** Smoothing: 1; Ring artefacts correction: 10; *L4 vertebrae* **-** Smoothing: 2; Ring artefacts correction: 10; *skulls* **-** Smoothing: 2; Ring artefacts correction: 15. Beam hardening correction was 35%. Grayscale thresholds were set between 75 and 255 for skull, ear and vertebrae images, and 95 to 255 for tibiae. All samples were realigned in DataViewer (version 1.5.4.0, Bruker) to ensure consistent sample orientation and µCT image segmentation and 2D/3D morphometric analyses were performed using CTAn (version 1.20.3.0, Bruker).

#### Skull

Cranial vertebrae were removed from aligned skull datasets using CTAn. DataViewer was used to measure average foramen magnum sagittal and transversal diameter, and parietal bone thickness between coronal/anterior lambdoid sutures (Fig. 4c). For morphometric skull analysis, cortical bone tissue volume (TV), bone volume (BV) and bone volume fraction (BV/TV), as well as trabecular TV, BV, BV/TV and thickness were measured. Middle ear otic capsule cortical parameters (tissue and bone area (T.Ar and B.Ar), bone area fraction (B.Ar/T.Ar)) and capsule thickness) were measured (Fig. 5a). BV, bone surface (BS), bone surface fraction (BS/BV) and ossicle thickness were also assessed.

#### Vertebrae

The cortical and trabecular bone between the superior and inferior end plates of the L4 lumbar vertebra were segmented (Fig. 6a). Disappearance of proximal/distal vertebral endplate spongiosa was set as reference, 10% offset applied and intervening trabecular/cortical parameters analysed.

#### Tibia

All bones except the tibia were removed from realigned datasets and the proximal metaphyseal trabecular bone was then segmented using the ‘trabecular bridge,’ where the two primary spongiosa ‘islands’ separated as a reference point. 5% of the total bone length from this point (away from diaphysis) was used for trabecular analysis (B.Ar, Tb.Sp, Tb.N and Tb.Th). Trabecular bone was segmented from the medullary area of the remaining tibial length, leaving only cortical bone for cortical analysis (TV, BV, BV/TV, B.Ar, T.Ar, B.Ar/T.Ar, tissue (T.Pm) and bone (B.Pm) perimeter).

### Immunohistochemistry

Paraffin embedded sections of L4 vertebrae were gradually rehydrated before overnight antigen retrieval with Uni-Trieve (CliniSciences, Slough, United Kingdom) at 40 °C according to manufacturer’s instructions. Sections were then permeabilised using 0.1% Triton-X, Tris-Buffered Saline (TBS) for 15 minutes at room temperature. Endogenous peroxidases were blocked using 0.3% hydrogen peroxide at 37 °C for 15 minutes prior to 3 × 5 minutes washes with wash buffer (0.1% TBS-Tween20). Sections were incubated in block buffer (10% Goat serum, 0.1% bovine serum albumin TBS) (Sigma-Aldrich) for 1 h at room temperature before washing 3 × 10 minutes with wash buffer, followed by overnight incubation at 4 °C with anti-AXIN2 antibody (1:200; Abcam 32197). Sections were washed 3 × 10 minutes in wash buffer before incubation with Goat anti-Rabbit HRP (1:200; Agilent P0448) for 1 h at room temperature, followed by 3 × 10 minute washes in wash buffer. Antibody staining was revealed using Signalstain® DAB Substrate Kit (Cell Signalling Technologies, Danvers, Massachusetts, USA). AXIN2 negative cells were revealed via counterstaining with Hoechst 33342 (5 µg/mL) for 10 minutes at room temperature before washing with wash buffer. Slides were imaged on a DMIRB inverted fluorescence microscope (Leica). The number of AXIN2-positive and negative osteocytes were manually counted using ImageJ to quantify the percentage of AXIN2-positive cells within the vertebral bone. It is important to note that DAB and Hoechst staining via this method is mutually exclusive, allowing direct quantification of positive and negative cells.

### Dynamic histomorphometry

Dynamic histomorphometry was performed by the University of Sheffield Skeletal Analysis Laboratories (University of Sheffield, UK). Labelled (with Alizarin Red: following loading/intermittent treatment; and Calcein: following further 2 weeks treatment only; inter label time = 7 days) loaded tibiae collected from male *Sost*^-/-^ mice treated with vehicle (*n* = 4) or LGK974 (*n* = 4) were fixed in 4% PFA for 48 h. Due to observed sexual dimorphism, male-loaded limbs were chosen for this as they likely represented the best opportunity to see changes in the labelled surfaces. Fixed samples were then stored in 70% ethanol in the dark until resin embedding. Tibiae samples were embedded in London Resin (LR) White and two 8 µm sections were cut and measured for each sample at two different levels, with 30 µm between levels. Bone and mineral parameters were measured using OsteoMeasure software (OsteoMetrics, Atlanta, GA, USA).

### Statistical analysis

In vitro data are presented as representative (≥3 technical replicates; mean ± SD). Data comparison was performed in GraphPad Prism 10 (Boston, Massachusetts, USA) using two-way ANOVA for bone formation assay, and unpaired t-tests or one-way ANOVA for trabecular and cortical parameters (skull, ears, and vertebra; mean ± SD). Whole bone 2D analyses of tibia cortical and trabecular compartments were completed in R version 4.2.3, using a linear mixed-effects model to assess the effects of LGK974 treatment, mechanical loading and their interaction at each distinct percentile along the tibia length. Shapiro-Wilk tests were performed to assess normality of residuals, and normality was assumed if most measures (10%–90%) along the tibia satisfied the null hypothesis of normality. Post-hoc comparisons between loaded and non-loaded effects within LGK974 treatment, or between LGK974 and vehicle effects within loading group were evaluated based on the estimated parameters from the mixed effects models. Data are shown as mean ± SEM and heatmaps that provide statistical significance (*P* < 0.001; 0.001 ≤ *P* < 0.01; 0.01 ≤ *P* < 0.05 and *P* ≥ 0.05) at each location were created. A *P*-value of <0.05 was considered significant.

## Supplementary information

Supplementary file 1

Supplementary file 2

## Data Availability

All the data support the figures, and the other findings are available upon reasonable request to the corresponding authors. Supplementary information accompanies the manuscript on the Bone Research website. Supplementary File S1 contains all supplementary figures and tables; Supplementary File 2 contains data pertaining to the RNAseq analysis.
