# Supplementary material for: Porcupine inhibition is a promising pharmacological treatment for severe sclerosteosis pathologies
Source: Bone Res. 2025 Apr 7;13:44. doi: 10.1038/s41413-025-00406-3 (PMC11973224; doi:10.1038/s41413-025-00406-3)
Supplement: Supplementary file 1 [file 41413_2025_406_MOESM1_ESM.docx]

**Supplementary materials**

Additional *in vitro* and *in vivo* methods and results expanding on method optimisation, results as well as conclusions made in the main article. Tables provide information about screened drugs, forward and reverse primers used for qPCR, and bone morphometric parameter data.

**Supplementary methods**

***In vitro* assessment of pamidronate disodium effect on mouse osteoclasts**

Pamidronate’s efficacy was validated using primary osteoclasts from *Sost*^-/-^ mice. Primary murine osteoclasts were prepared as described previously^1^. Briefly, bone marrow flushed from 6-week-old C57/BL6 and *Sost*^-/-^ mouse long bones using DPBS, cultured for 24 hours in the presence of 50 ng/mL macrophage colony stimulating factor (M-CSF) (Peprotech) and 100 nM prostaglandin E2 (PGE2) (Sigma-Aldrich) in MEM containing 10% FBS, 2 mM L-glutamine, 100 U/mL penicillin, 100 μg/mL streptomycin and 0.25 μg/mL amphotericin. After 24 hours, cells were seeded onto dentine discs (1x10^6^ cells/5 mm disc) and allowed to adhere for 24 hours in MEM containing 200 ng/mL M-CSF, 5 ng/mL RANK-L (Peprotech) and 100 nM PGE2. Discs were then transferred to 6-well plates (4 discs/well) and maintained in varying concentrations of pamidronate disodium (APExBIO) for 3 days. On day 5, 12 M HCl was added to adjust medium pH to 7.0, facilitating initiation of resorption. Treatment with/without pamidronate disodium was continued to day 7 and cells then fixed in 2.5% glutaraldehyde. Fixed osteoclasts were stained for tartrate-resistant acid phosphatase (TRAP) and dentine discs imaged by transmitted light microscopy. Images were processed through an automated counting method, using the Fiji Ilastik plugin^2-4^. Resorption pits/disc were counted blindly in a transmitted light microscope image with a dot-counting morphometry system using Fiji^1^.

***In vivo* evaluation of pamidronate disodium treatment in *Sost*^-/-^ mice**

Male *Sost*^-/-^ mice (6-week-old; n = 10) were housed in polypropylene cages with environmental enrichment and a 12 h light/dark cycle at 21±2°C. Mice had access to RM1 standard diet (LBS Biotechnology) and water *ad libitum*. Each mouse was weighed prior to dosing. Mice (n = 5 animals in vehicle group; n = 5 in pamidronate disodium treatment group) were subjected to the loading regimen (described in main manuscript), with 2x/week subcutaneous dosing of either vehicle (PBS) or 2 mg/kg pamidronate disodium for 2 weeks^5^. Mice were then maintained for 6 weeks and culled. Bone samples were fixed, and microarchitecture of the tibia and vertebrae was analysed by microcomputed tomography (µCT) using CTAn (Bruker, Belgium), as described in the main manuscript.

**Supplementary figures**


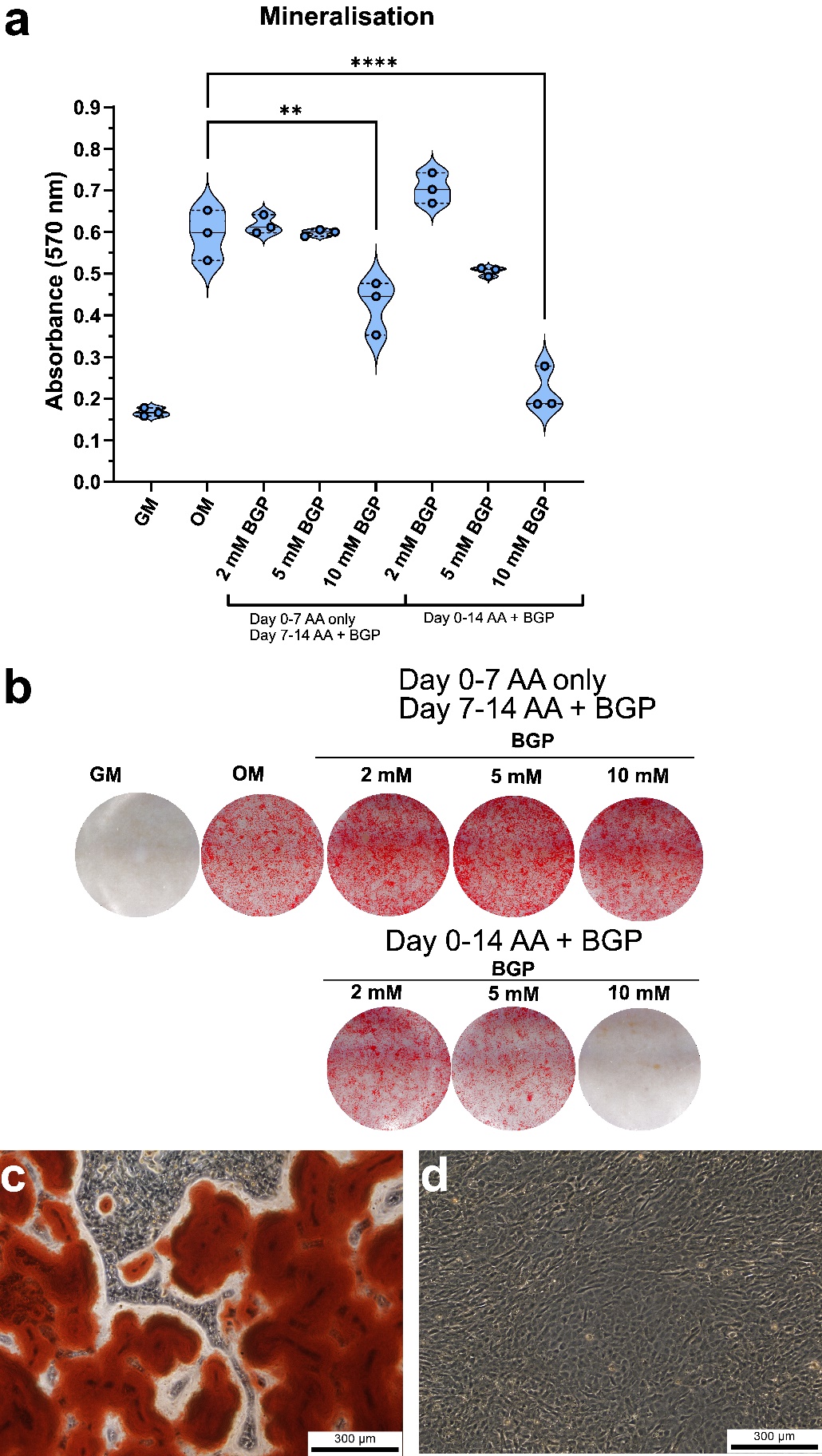


**Supplementary figure 1: Optimisation of osteoblast-like cell growth and mineralisation conditions.** (**a**) Osteoblast (MC3T3-E1) mineralisation at day 14 as measured by Alizarin Red S and CPC. (**b**) Representative whole well scans of osteoblast cell layers cultured in different conditions are shown below the mineralisation graph. Different conditions assessed: growth media (alpha MEM; GM) supplemented with 0 (OM), 2, 5 and 10 mM β-glycerophosphate (BGP) from day 0 to 14 or from day 7 to 14, and 50 ​μg/mL ascorbate-2-phosphate (AA2P) from day 0. (**c**) Representative light microscope image of osteoid and ARS-stained mineralised matrix at day 14 (cells cultured in GM supplemented with 2 mM BGP from day 7 to 14. (**d**) Representative light microscope image of control osteoblasts cultured in GM only. In the graph, means of each group are indicated by the solid line, and upper and lower dashed lines represent quartiles. ** p ≤ 0.01 and **** p ≤ 0.0001.


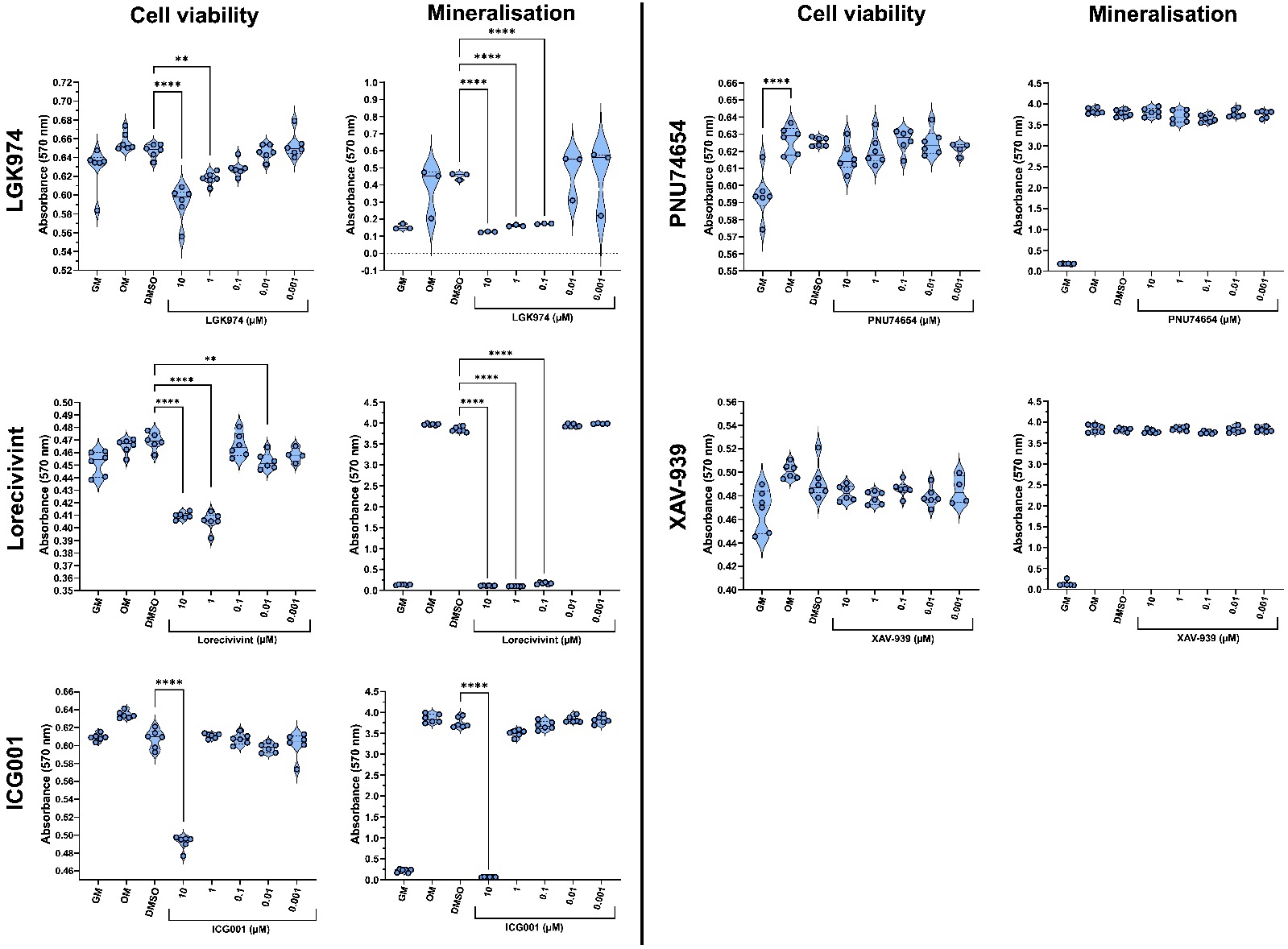


**Supplementary figure 2: Candidate inhibitor screen in osteoblast-like cells.** MC3T3-E1 cells were treated with various concentrations (10, 1, 0.1, 0.01 and 0.001 μM) of selected therapeutics (LGK974, Lorecivivint, ICG001, PNU74654 and XAV-939). Control cells were cultured in growth media (GM). Osteogenic conditions (OM): GM supplemented with 50 ​μg/mL AA (Day 0 – Day 14) and 2 mM BGP (Day 7 – Day 14). Drug effect on cell viability and mineralisation were assessed on day 14. For all graphs, means of each group are indicated by the solid line, and upper and lower dashed lines represent quartiles. ** p ≤ 0.01 and **** p ≤ 0.0001.


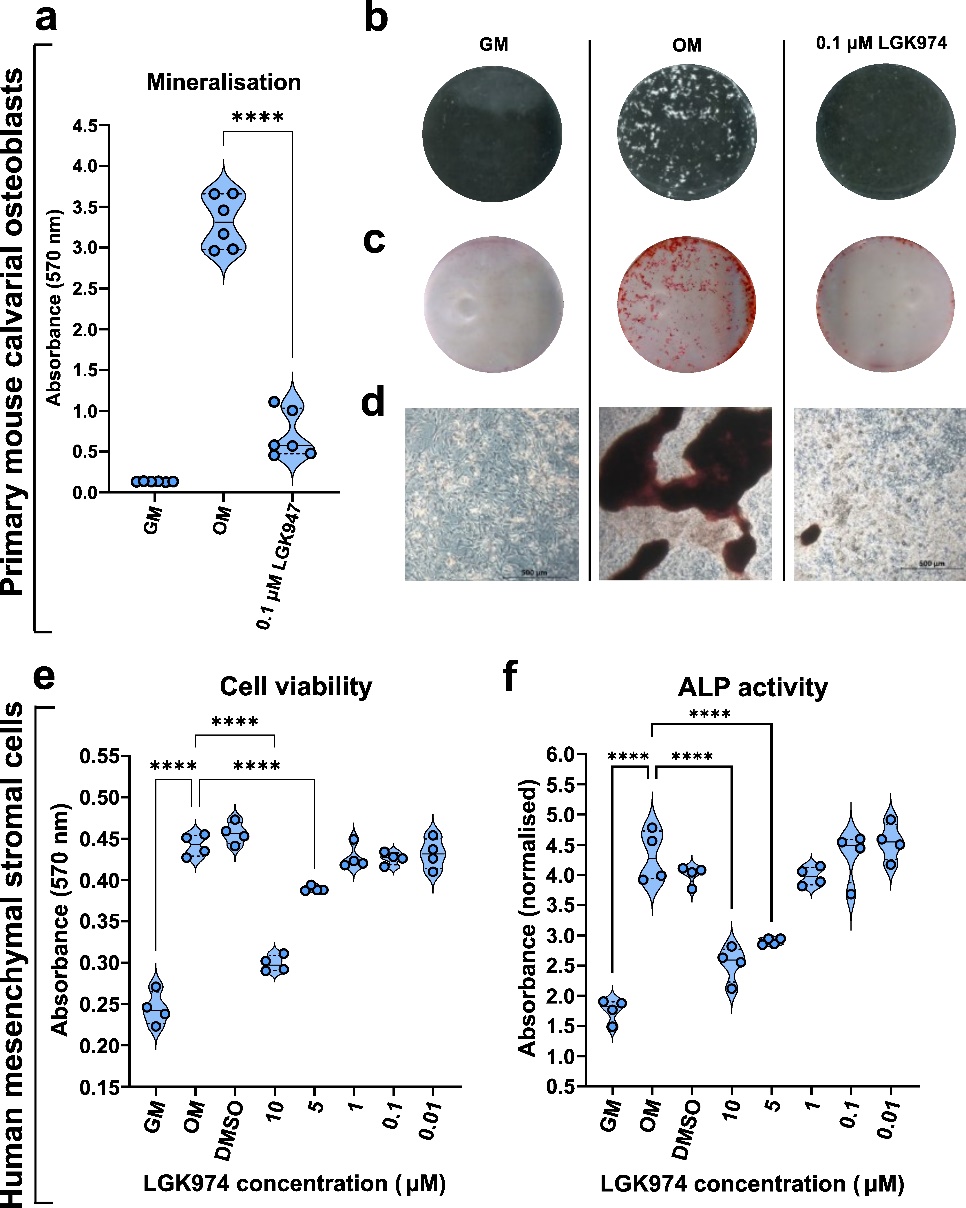


**Supplementary figure 3: LGK974 reduces activity and mineralisation of primary mouse calvarial osteoblasts and human mesenchymal stromal cells.** (**a**) LGK974 treatment reduces mineralisation of primary mouse calvarial osteoblasts after 21 days. (**b**) Representative whole well scans of non-stained, fixed primary osteoblast cell layers. Mineralisation is visible in OM treated primary osteoblasts after 21 days. (**c**) Representative whole well scans of Alizarin Red S (ARS) stained primary osteoblast cell layers. (**d**) Representative light microscope images of primary mouse calvarial osteoblasts stained with ARS at day 21. (**e**) Effect of dose dependent LGK974 treatment on human mesenchymal stromal cell (hMSC) viability and (**f**) ALP activity at day 21. In the graphs, means of each group are indicated by the solid line, and upper and lower dashed lines represent quartiles. *** p ≤ 0.001 and **** p ≤ 0.0001.


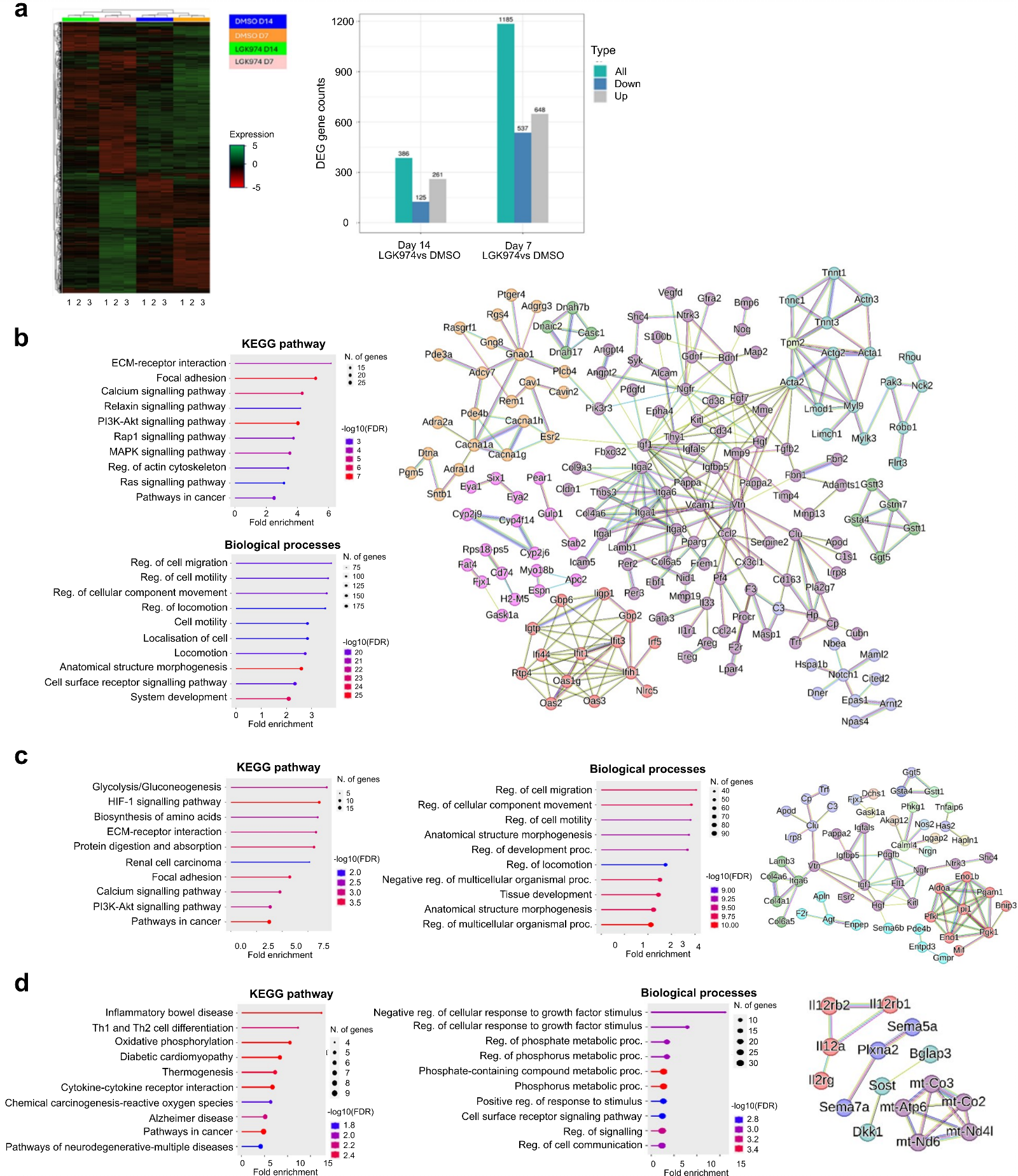


**Supplementary figure 4: Protein-protein interaction network and pathway enrichment analysis from LGK974 treated MC3T3-osteoblast like cell bulk-RNAseq.** (**a**) Total differential gene expression heatmap and DEG count across conditions and timepoints (n = 3/group) shows LGK974 to have the greatest effect on gene expression on day 7. (**b**) Day 7 upregulated; (**c**) Day 14 upregulated gene expression; (**d**) Day 14 downregulated DEGs and STRING protein-protein interaction and pathway enrichment analysis.


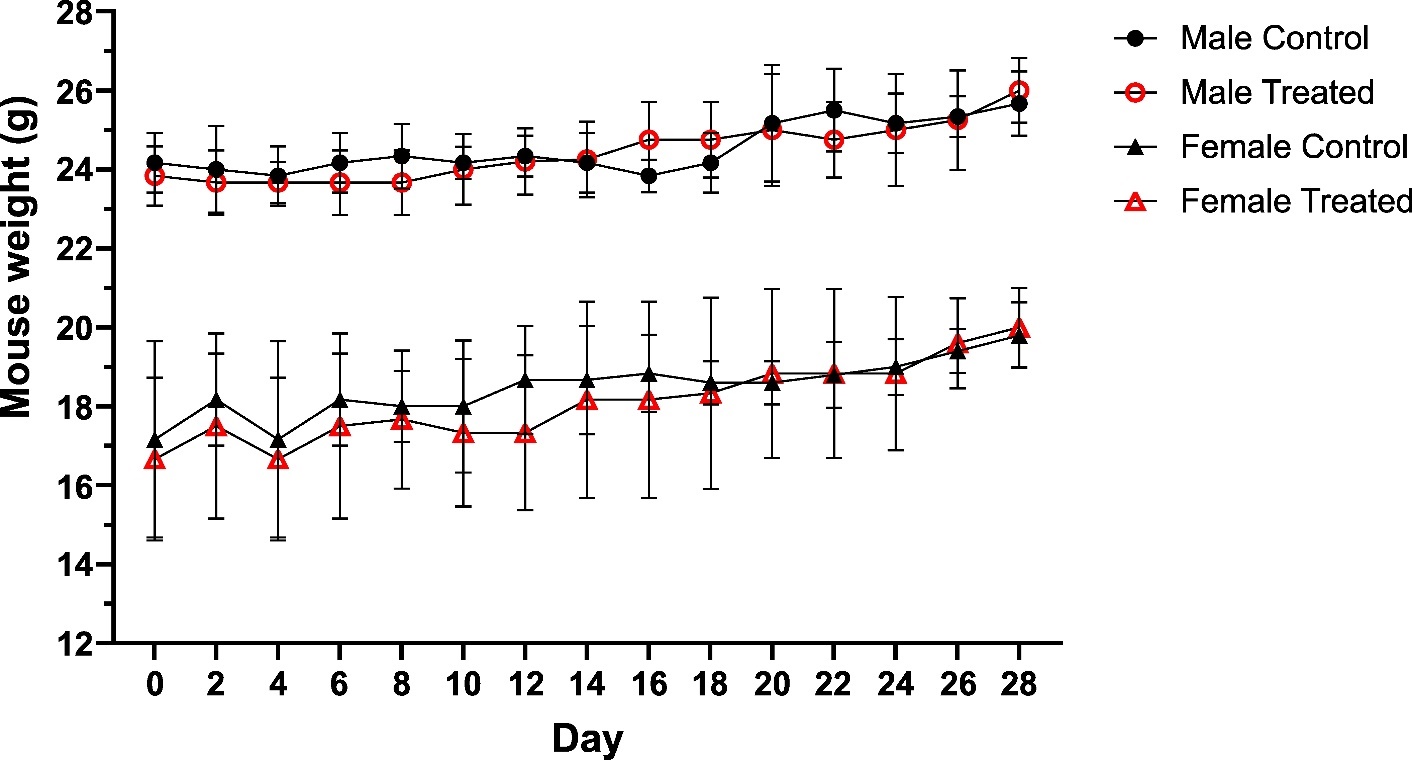


**Supplementary figure 5: LGK974 treatment has no effect on *Sost*^-/-^ mouse weight.** Body weights of male and female treated and untreated *Sost*^-/-^ mice. Black solid circles = male vehicle controls; red circle = male LGK974 treated; black solid triangle = female vehicle controls; red triangle = female LGK974 treated groups. Group sizes were *n* = 5 treated females, *n* = 6 non-treated females, *n* = 4 treated males and *n* = 5 non-treated males.


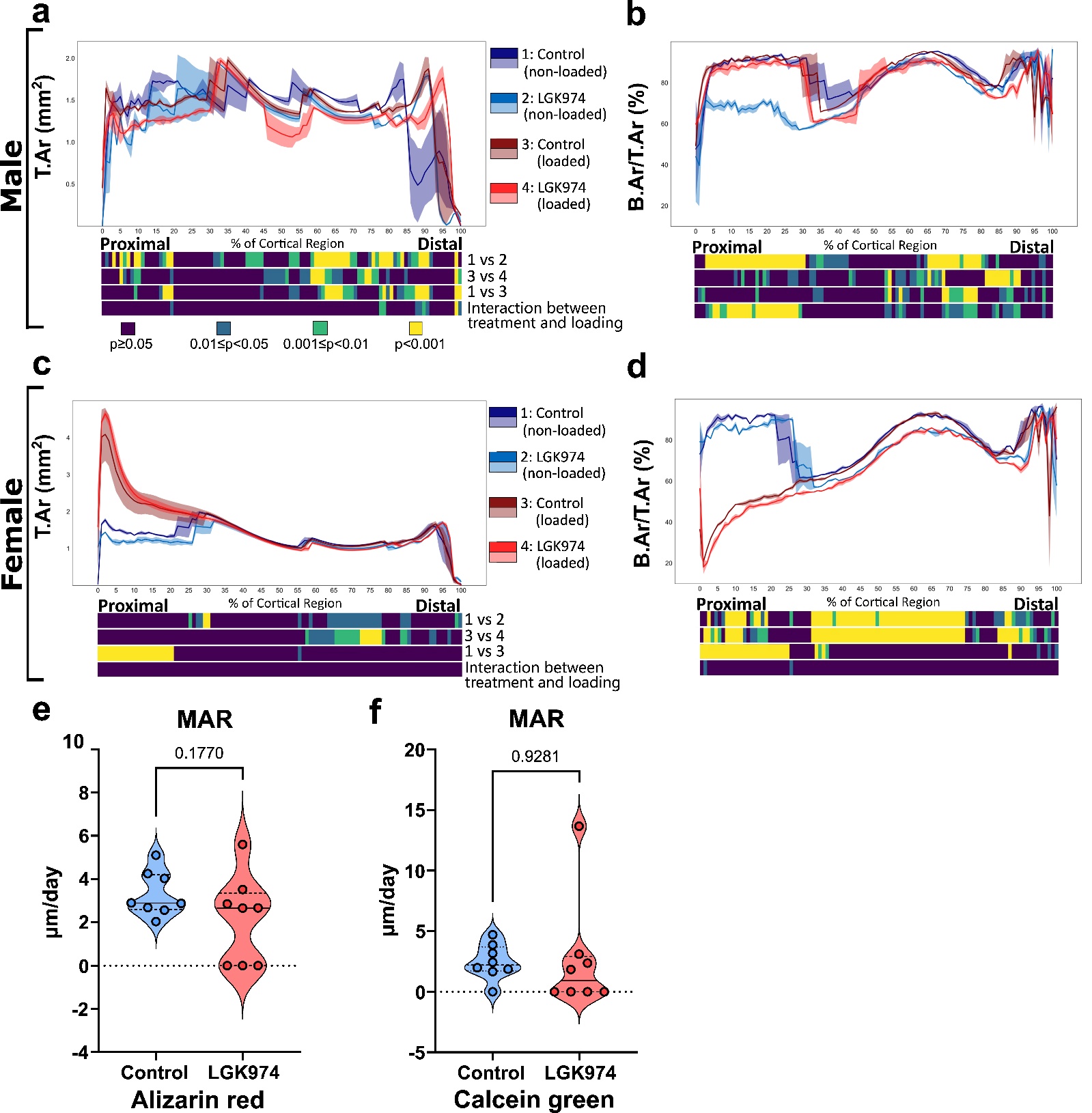


**Supplementary figure 6:** **LGK974 reduces tibial cortical bone morphometric parameters in *Sost*^-/-^ mice.** Effect of LGK974 on cortical (**a**) T.Ar and (**b**) bone area percentage (B,Ar/T.Ar) of male loaded and non-loaded tibiae. (**c** and **d**) Effect of LGK974 on the same cortical parameters of female tibiae. (**e**) Mineral apposition rate (MAR) of control and LGK974 treated male loaded tibia (alizarin red dual labelled; day 4 and 11). Effect of LGK974 on (**f**) MAR of male loaded tibia towards the end of the study (calcein dual labelled; day 18 and 25). Line graphs represent means ± SEM (1 = non-loaded control (dark blue); 2 = non-loaded LGK974 treated (light blue); 3 = loaded control (maroon); 4 = loaded LGK974 treated (red)). Graphical heat maps below the graphs summarises statistical differences at specific matched locations along the length of the tibiae and represent the overall effect of LGK974 treatment (dark blue p ≥ 0.05, blue 0.01 ≤ p < 0.05, green 0.001 ≤ p < 0.01, yellow p < 0.001). Group sizes were *n* = 5 treated females, *n* = 6 non-treated females, *n* = 4 treated males and *n* = 5 non-treated males. For dynamic histomorphometry violin plots, means of each group (group sizes were *n* = 4 control and *n* = 4 treated males, with two levels analysed for each tibia) are indicated by the solid line, and upper and lower dashed lines represent quartiles. Blue and red represent vehicle controls and LGK974 treated groups respectively.


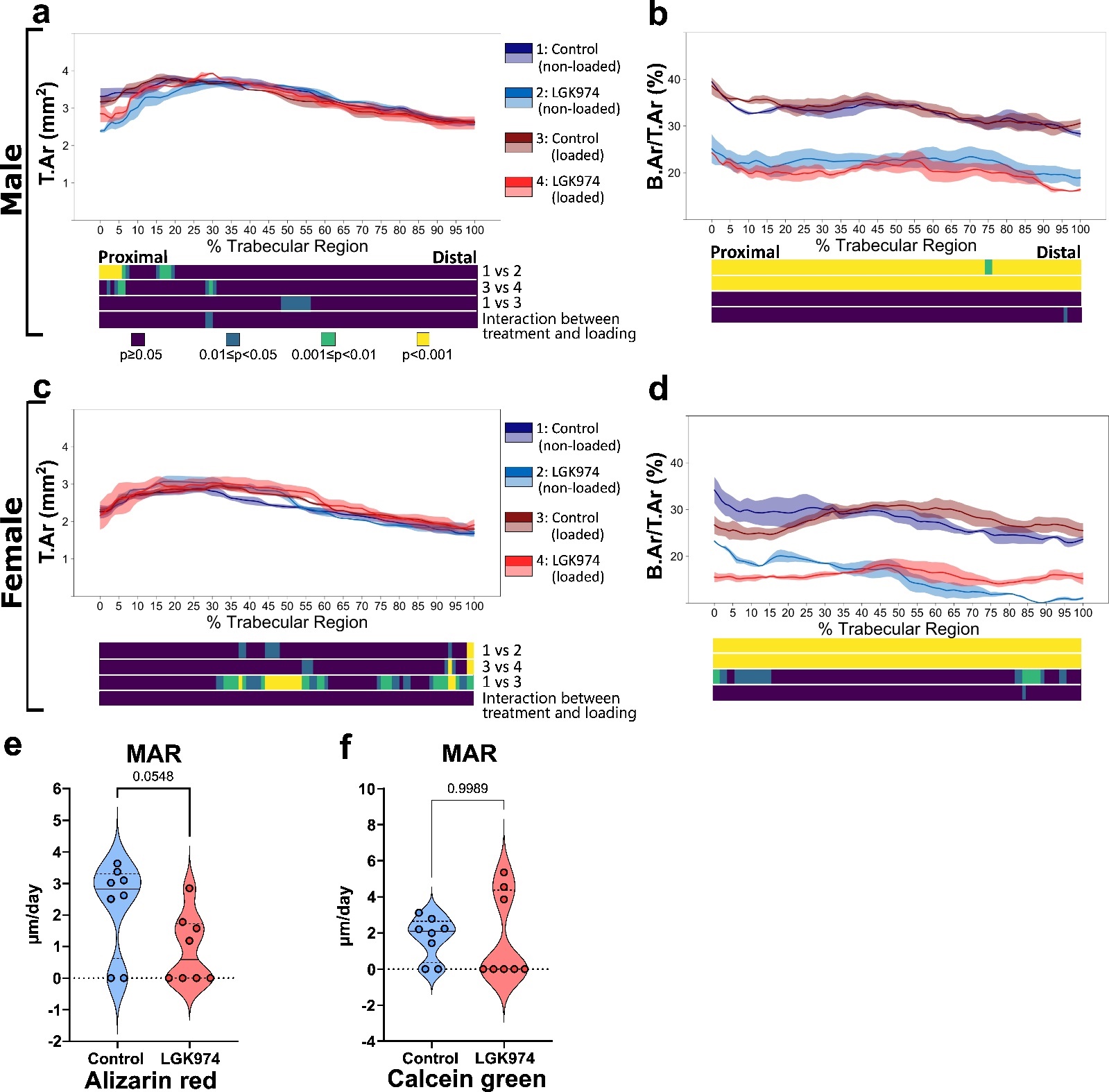


**Supplementary figure 7:** **LGK974 reduces tibial trabecular bone morphometric parameters in *Sost*^-/-^ mice.** Effect of LGK974 on trabecular (**a**) T.Ar and (**b**) bone area percentage (B,Ar/T.Ar) of male loaded and non-loaded tibiae. (**c** and **d**) Effect of LGK974 on the same trabecular parameters of female tibiae. (**e**) Mineral apposition rate (MAR) of control and LGK974 treated male loaded tibia (alizarin red dual labelled; day 4 and 11). Effect of LGK974 on (**f**) MAR of male loaded tibia towards the end of the study (calcein dual labelled; day 18 and 25). Line graphs represent means ± SEM (1 = non-loaded control (dark blue); 2 = non-loaded LGK974 treated (light blue); 3 = loaded control (maroon); 4 = loaded LGK974 treated (red)). Graphical heat maps below the graphs summarises statistical differences at specific matched locations along the length of the tibiae and represent the overall effect of LGK974 treatment (dark blue p ≥ 0.05, blue 0.01 ≤ p < 0.05, green 0.001 ≤ p < 0.01, yellow p < 0.001). Group sizes were *n* = 5 treated females, *n* = 6 non-treated females, *n* = 4 treated males and *n* = 5 non-treated males. For dynamic histomorphometry violin plots, means of each group (group sizes were *n* = 4 control and *n* = 4 treated males, with two levels analysed for each tibia) are indicated by the solid line, and upper and lower dashed lines represent quartiles. Blue and red represent vehicle controls and LGK974 treated groups respectively. *** p ≤ 0.001.


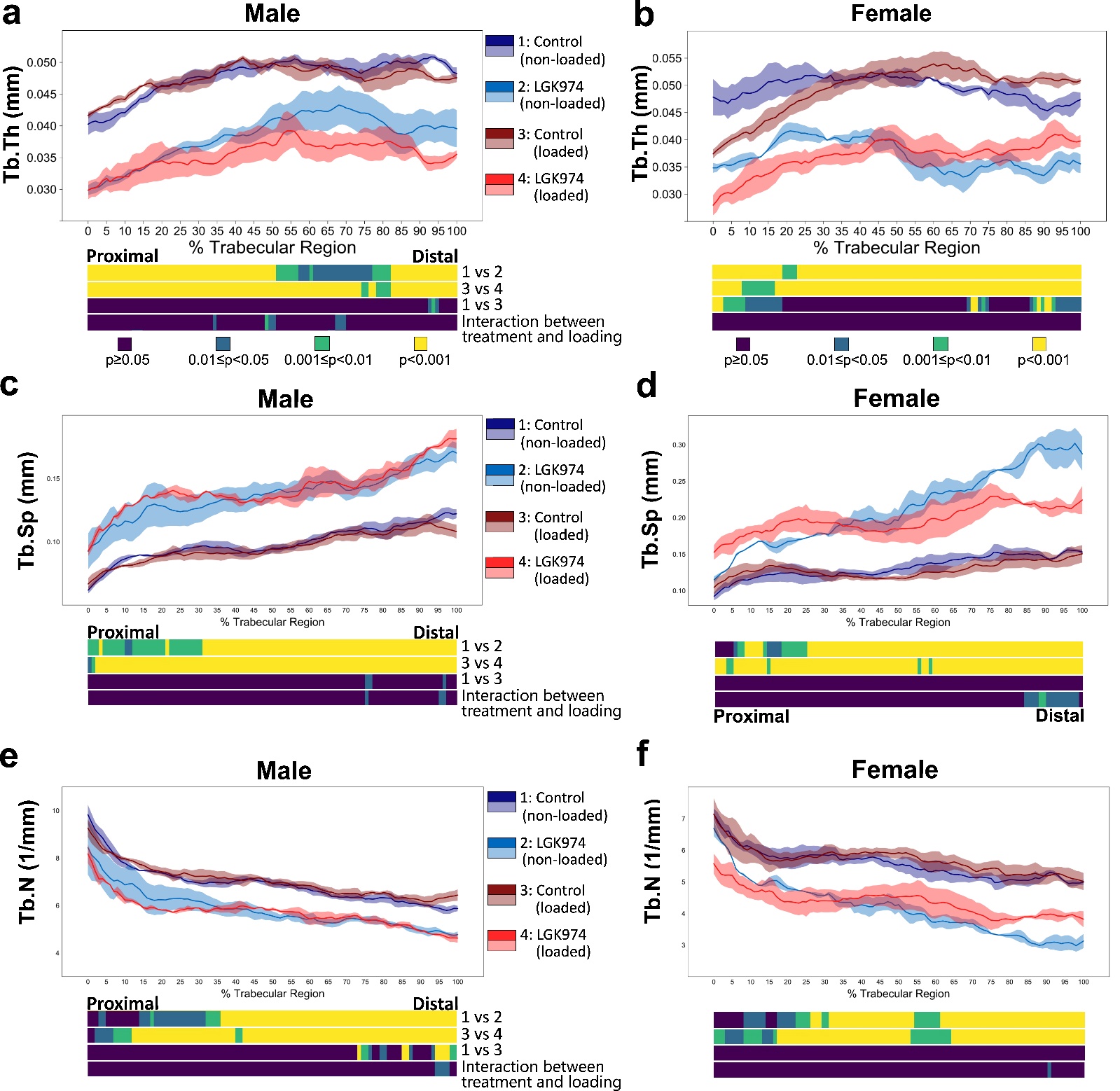


**Supplementary figure 8:** **LGK974 reduces tibial trabecular bone morphometric parameters in *Sost*^-/-^ mice.** Effect of LGK974 on (**a and b**) trabecular thickness (Tb.Th), (**c and d**), space (Tb.Sp) and (**e and f**) number (Tb.N) of male and female loaded and non-loaded tibiae. Line graphs represent means ± SEM (1 = non-loaded control (dark blue); 2 = non-loaded LGK974 treated (light blue); 3 = loaded control (maroon); 4 = loaded LGK974 treated (red)). Graphical heat maps below the graphs summarises statistical differences at specific matched locations along the length of the tibiae and represent the overall effect of LGK974 treatment (dark blue p ≥ 0.05, blue 0.01 ≤ p < 0.05, green 0.001 ≤ p < 0.01, yellow p < 0.001). Group sizes were *n* = 5 treated females, *n* = 6 non-treated females, *n* = 4 treated males and *n* = 5 non-treated males.


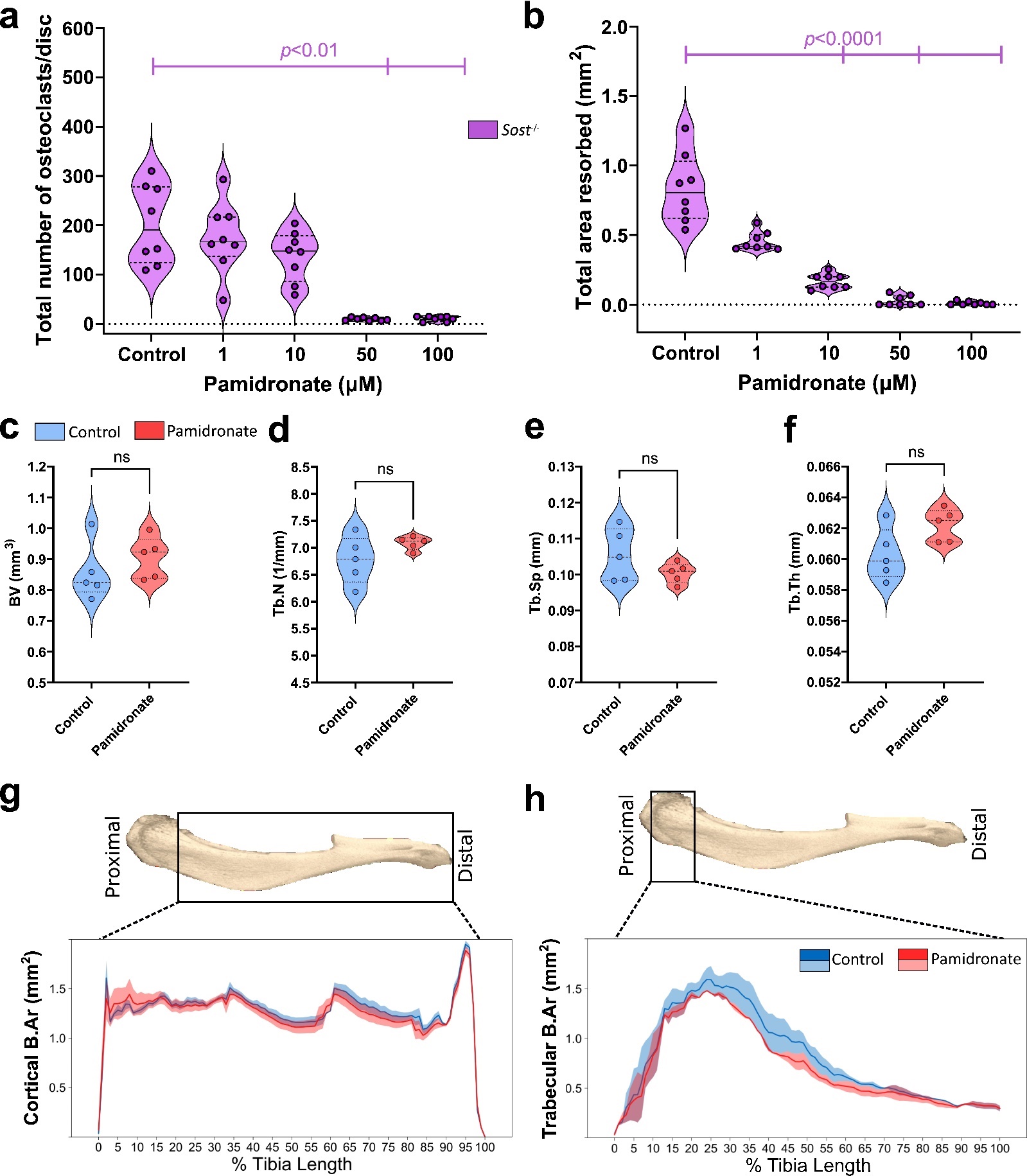


**Supplementary figure 9: Pamidronate disodium modifies *Sost*^-/-^ mouse osteoclast parameters, but has no effect on vertebral and tibial bone.** (**a**) Total number of *Sost*^-/-^ TRAP+ mature osteoclasts on dentine discs after 7 days of culture and treatment with pamidronate disodium (1 to 100 μM) were assessed using the Fiji Ilastik plugin. (**b**) Total area resorbed by osteoclasts was calculated using an automated counting method in Fiji. (**c**) Effect of pamidronate disodium treatment on L4 lumbar vertebral body BV, (**d**) trabecular number (Tb.N), (**e**) space (Tb.Sp) and (**f**) thickness (Tb.Th). (**g**) Effect of pamidronate disodium on tibial cortical and (**h**) trabecular bone area (B.Ar). For violin plots, means of each group are indicated by a solid line, and upper and lower dashed lines represent quartiles. Purple represents osteoclasts from *Sost*^-/-^ mice (n = 8 for each group). Blue represents the vehicle control group, whilst red represents the pamidronate disodium treated group. Line graphs represent means ± SEM (control = blue; pamidronate disodium treated = red). Group sizes were *n* = 5 treated males and *n* = 5 non-treated males.

**Supplementary Table 1: Screened small molecule inhibitors**

| **Drug** | **Wnt/B-catenin pathway target node** | **References** |
| --- | --- | --- |
| LGK974 | PORCN | Funck-Brentano *et al.*, 2018, Madan *et al.*, 2018 |
| ICG001 | CBP | Chung *et al.*, 2013, Gravesen *et al.*, 2018) |
| PNU74654 | TCF4 | Trosset *et al.*, 2006, Chen *et al.*, 2020 |
| lorecivivint | Indirectly targets GSK3β | Deshmukh *et al.*, 2020 |
| XAV939 | TNKS | Bao *et al.*, 2012, Fujita *et al.*, 2018, Li *et al.*, 2018 |

**Supplementary Table 2: Primer sequences**

| **Primer** | **Sequence (5'->3')** |
| --- | --- |
| **Axin2** |  |
| Forward primer | AAGCAGCCGTTCGCGATG |
| Reverse primer | CTCATGTGAGCCTCCTCTCTTT |
|  |  |
| **Runx2** |  |
| Forward primer | AGTCCATGCAGGAATATTTAAGGC |
| Reverse primer | AGGCTGTTTGACGCCATAGT |
|  |  |
| **Ocn** |  |
| Forward primer | CCCTGAGTCTGACAAAGCCTTC |
| Reverse primer | GTAGCGCCGGAGTCTGTTC |
|  |  |
| **Col1a** |  |
| Forward primer | CGATGGATTCCCGTTCGAGT |
| Forward primer | GAGGCCTCGGTGGACATTAG |
|  |  |
| **Trap** |  |
| Forward primer | ATTTGTGGCTGTGGGCGACT |
| Reverse primer | GCACGGTTCTGGCGATCTCT |
|  |  |

**Supplementary Table 3: *Sost*^-/-^ bone morphometric parameter data (LGK974 and vehicle treated)**

| **Tissue parameters** | | **Sex** | **Group** | **Mean** | **±** | **SD** | **Percentage change (%)** | | |
| --- | --- | --- | --- | --- | --- | --- | --- | --- | --- |
| **Skull** | |  |  |  |  |  |  |  | |
| TV | | Male | Vehicle | 1159.05 | ± | 49.8859 | -3.39 |  | |
|  |  |  | LGK974 | 1119.76 | ± | 25.5960 |  |  |  |
|  |  | Female | Vehicle | 1111.50 | ± | 48.0359 | 0.92 |  | |
|  |  |  | LGK974 | 1121.76 | ± | 42.9735 |  |  |  |
| BV | | Male | Vehicle | 202.17 | ± | 10.5890 | -11.60 | ** | |
|  |  |  | LGK974 | 178.71 | ± | 7.9129 |  |  | |
|  |  | Female | Vehicle | 197.08 | ± | 14.9687 | -4.80 |  | |
|  |  |  | LGK974 | 187.62 | ± | 10.4416 |  |  | |
| BV/TV | | Male | Vehicle | 17.44 | ± | 0.2037 | -8.44 | ** | |
|  |  |  | LGK974 | 15.96 | ± | 0.5225 |  |  | |
|  |  | Female | Vehicle | 17.72 | ± | 1.0150 | -5.63 |  | |
|  |  |  | LGK974 | 16.72 | ± | 0.3233 |  |  | |
| Parietal thickness (left) Tb.Th (pl) | | Male | Vehicle | 0.0238 | ± | 0.0003 | -1.14 |  | |
|  |  |  | LGK974 | 0.0236 | ± | 0.0001 |  |  | |
|  |  | Female | Vehicle | 0.0237 | ± | 0.0004 | -0.73 |  | |
|  |  |  | LGK974 | 0.0235 | ± | 0.0004 |  |  | |
| Parietal thickness (right) Tb.Th (pl) | | Male | Vehicle | 0.0237 | ± | 0.0003 | -2.39 |  | |
|  |  |  | LGK974 | 0.0231 | ± | 0.0007 |  |  | |
|  |  | Female | Vehicle | 0.0238 | ± | 0.0004 | -0.79 |  | |
|  |  |  | LGK974 | 0.0236 | ± | 0.0003 |  |  | |
| Foramen magnum diameter (sagital) | | Male | Vehicle | 3532.08 | ± | 22.5062 | 0.99 |  | |
|  |  |  | LGK974 | 3567.05 | ± | 34.7278 |  |  | |
|  |  | Female | Vehicle | 3486.27 | ± | 111.3122 | 0.85 |  | |
|  |  |  | LGK974 | 3515.90 | ± | 70.1965 |  |  | |
| Foramen magnum diameter (transversal) | | Male | Vehicle | 5028.88 | ± | 39.9430 | -1.63 |  | |
|  |  |  | LGK974 | 4946.78 | ± | 101.6247 |  |  | |
|  |  | Female | Vehicle | 4953.50 | ± | 61.4028 | 0.11 |  | |
|  |  |  | LGK974 | 4958.90 | ± | 51.7796 |  |  | |
|  | |  |  |  |  |  |  |  | |
| **Vertebrae** | |  |  |  |  |  |  |  | |
| TV | | Male | Vehicle | 1.46 | ± | 0.1720 | -4.89 |  | |
|  |  |  | LGK974 | 1.39 | ± | 0.0208 |  |  | |
|  |  | Female | Vehicle | 1.27 | ± | 0.0871 | 10.45 |  | |
|  |  |  | LGK974 | 1.40 | ± | 0.1514 |  |  | |
| BV | | Male | Vehicle | 0.61 | ± | 0.0803 | -22.84 | * | |
|  |  |  | LGK974 | 0.47 | ± | 0.0390 |  |  | |
|  |  | Female | Vehicle | 0.53 | ± | 0.0677 | -15.46 |  | |
|  |  |  | LGK974 | 0.45 | ± | 0.0728 |  |  | |
| BV/TV | | Male | Vehicle | 41.78 | ± | 1.7278 | -18.75 | ** | |
|  |  |  | LGK974 | 33.95 | ± | 3.0498 |  |  | |
|  |  | Female | Vehicle | 41.75 | ± | 3.7542 | -23.56 | *** | |
|  |  |  | LGK974 | 31.92 | ± | 2.7671 |  |  | |
| Tb.Th | | Male | Vehicle | 0.0608 | ± | 0.0030 | 1.35 |  | |
|  |  |  | LGK974 | 0.0617 | ± | 0.0023 |  |  | |
|  |  | Female | Vehicle | 0.0605 | ± | 0.0049 | -5.97 |  | |
|  |  |  | LGK974 | 0.0569 | ± | 0.0033 |  |  | |
| Tb.N | | Male | Vehicle | 6.87 | ± | 0.1018 | -19.90 | ** | |
|  |  |  | LGK974 | 5.50 | ± | 0.3952 |  |  | |
|  |  | Female | Vehicle | 6.90 | ± | 0.3166 | -18.74 | **** | |
|  |  |  | LGK974 | 5.61 | ± | 0.3062 |  |  | |
| Tb.Sp | | Male | Vehicle | 0.108 | ± | 0.0022 | 24.26 | ** | |
|  |  |  | LGK974 | 0.134 | ± | 0.0079 |  |  | |
|  |  | Female | Vehicle | 0.110 | ± | 0.0070 | 23.47 | ** | |
|  |  |  | LGK974 | 0.136 | ± | 0.0111 |  |  | |
|  | |  |  |  |  |  |  |  | |
| **Otic capsule** | |  |  |  |  |  |  |  | |
| B.Ar | | Male | Vehicle | 1.18 | ± | 0.0476 | -7.77 | * | |
|  |  |  | LGK974 | 1.09 | ± | 0.0471 |  |  | |
|  |  | Female | Vehicle | 1.12 | ± | 0.0421 | -1.28 |  | |
|  |  |  | LGK974 | 1.11 | ± | 0.0654 |  |  | |
| T.Ar | | Male | Vehicle | 2.74 | ± | 0.0865 | -5.62 | * | |
|  |  |  | LGK974 | 2.58 | ± | 0.0773 |  |  | |
|  |  | Female | Vehicle | 2.64 | ± | 0.0558 | -1.43 |  | |
|  |  |  | LGK974 | 2.60 | ± | 0.0835 |  |  | |
| B.Ar/T.Ar | | Male | Vehicle | 43.09 | ± | 0.8906 | -2.22 |  | |
|  |  |  | LGK974 | 42.13 | ± | 0.5522 |  |  | |
|  |  | Female | Vehicle | 42.60 | ± | 0.7279 | 0.11 |  | |
|  |  |  | LGK974 | 42.65 | ± | 1.2663 |  |  | |
| Otic capsule thickness | | Male | Vehicle | 0.0236 | ± | 0.0001 | -0.62 | * | |
|  |  |  | LGK974 | 0.0235 | ± | 0.0001 |  |  | |
|  |  | Female | Vehicle | 0.0235 | ± | 0.0001 | 0.12 |  | |
|  |  |  | LGK974 | 0.0236 | ± | 0.0001 |  |  | |
|  | |  |  |  |  |  |  |  | |
| **Ossicles** | |  |  |  |  |  |  |  | |
| BV | | Male | Vehicle | 0.216 | ± | 0.0059 | -7.99 | * | |
|  |  |  | LGK974 | 0.198 | ± | 0.0092 |  |  | |
|  |  | Female | Vehicle | 0.199 | ± | 0.0079 | -2.20 |  | |
|  |  |  | LGK974 | 0.195 | ± | 0.0099 |  |  | |
| BS | | Male | Vehicle | 5.70 | ± | 0.2779 | -11.11 | ** | |
|  |  |  | LGK974 | 5.07 | ± | 0.2281 |  |  | |
|  |  | Female | Vehicle | 5.24 | ± | 0.1860 | -4.24 |  | |
|  |  |  | LGK974 | 5.02 | ± | 0.1431 |  |  | |
| BS/BV | | Male | Vehicle | 26.44 | ± | 0.8752 | -3.34 |  | |
|  |  |  | LGK974 | 25.56 | ± | 0.8185 |  |  | |
|  |  | Female | Vehicle | 26.34 | ± | 1.1524 | -2.08 |  | |
|  |  |  | LGK974 | 25.79 | ± | 0.6654 |  |  | |
| Ossicle thickness | | Male | Vehicle | 0.202 | ± | 0.0106 | -1.73 |  | |
|  |  |  | LGK974 | 0.198 | ± | 0.0086 |  |  | |
|  |  | Female | Vehicle | 0.196 | ± | 0.0121 | 2.89 |  | |
|  |  |  | LGK974 | 0.201 | ± | 0.0092 |  |  | |
|  | |  |  |  |  |  |  |  | |
| **Tibia (cortical; loaded)** | |  |  |  |  |  |  |  | |
| TV | | Male | Vehicle | 24.03 | ± | 0.1213 | -11.72 | ** | |
|  |  |  | LGK974 | 21.21 | ± | 0.6122 |  |  | |
|  |  | Female | Vehicle | 27.79 | ± | 0.4247 | -11.66 | *** | |
|  |  |  | LGK974 | 24.55 | ± | 0.7358 |  |  | |
| BV | | Male | Vehicle | 20.95 | ± | 0.2336 | -18.10 | *** | |
|  |  |  | LGK974 | 17.16 | ± | 0.5469 |  |  | |
|  |  | Female | Vehicle | 16.45 | ± | 1.1238 | -15.73 | ** | |
|  |  |  | LGK974 | 13.86 | ± | 0.1959 |  |  | |
| BV/TV | | Male | Vehicle | 87.19 | ± | 1.0849 | -7.24 | **** | |
|  |  |  | LGK974 | 80.88 | ± | 0.5716 |  |  | |
|  |  | Female | Vehicle | 59.17 | ± | 3.5984 | -4.52 |  | |
|  |  |  | LGK974 | 56.49 | ± | 1.4231 |  |  | |
|  | |  |  |  |  |  |  |  | |
| **Tibia (cortical; non-loaded)** | |  |  |  |  |  |  |  | |
| TV | | Male | Vehicle | 23.51 | ± | 0.3341 | -8.46 | *** | |
|  |  |  | LGK974 | 21.53 | ± | 0.4347 |  |  | |
|  |  | Female | Vehicle | 21.20 | ± | 0.3524 | -11.12 | ** | |
|  |  |  | LGK974 | 18.84 | ± | 0.8605 |  |  | |
| BV | | Male | Vehicle | 21.17 | ± | 0.5229 | -17.15 | **** | |
|  |  |  | LGK974 | 17.54 | ± | 0.3132 |  |  | |
|  |  | Female | Vehicle | 17.10 | ± | 0.1859 | -17.31 | ** | |
|  |  |  | LGK974 | 14.14 | ± | 0.9805 |  |  | |
| BV/TV | | Male | Vehicle | 90.03 | ± | 2.1454 | -8.86 | *** | |
|  |  |  | LGK974 | 82.05 | ± | 1.6965 |  |  | |
|  |  | Female | Vehicle | 80.70 | ± | 1.3374 | -7.03 | *** | |
|  |  |  | LGK974 | 75.02 | ± | 2.6457 |  |  | |
|  | |  |  |  |  |  |  |  | |
| **Tibia (trabecular; loaded)** |  |  |  |  |  |  | |  |  |
| TV | Male | Vehicle | 3.84 | ± | 0.3075 | -21.79 | | ** |  |
|  |  | LGK974 | 3.01 | ± | 0.1881 |  |  |  |  |
|  | Female | Vehicle | 3.95 | ± | 0.1537 | -16.62 | | ** |  |
|  |  | LGK974 | 3.29 | ± | 0.3487 |  |  |  |  |
| BV | Male | Vehicle | 0.53 | ± | 0.0218 | -14.50 | | ** |  |
|  |  | LGK974 | 0.45 | ± | 0.0327 |  |  |  |  |
|  | Female | Vehicle | 0.51 | ± | 0.0306 | -13.72 | | *** |  |
|  |  | LGK974 | 0.44 | ± | 0.0286 |  |  |  |  |
|  |  |  |  |  |  |  | |  |  |
| **Tibia (trabecular; non-loaded)** |  |  |  |  |  |  | |  |  |
| TV | Male | Vehicle | 4.17 | ± | 0.4879 | -27.17 | | *** |  |
|  |  | LGK974 | 3.04 | ± | 0.3401 |  |  |  |  |
|  | Female | Vehicle | 3.80 | ± | 0.3988 | -7.21 | | *** |  |
|  |  | LGK974 | 3.53 | ± | 0.3290 |  |  |  |  |
| BV | Male | Vehicle | 0.49 | ± | 0.0333 | -20.44 | | *** |  |
|  |  | LGK974 | 0.39 | ± | 0.0330 |  |  |  |  |
|  | Female | Vehicle | 0.40 | ± | 0.0231 | -21.69 | |  |  |
|  |  | LGK974 | 0.31 | ± | 0.0259 |  |  |  |  |

Statistical comparisons: * p ≤ 0.05, ** p ≤ 0.01, *** p ≤ 0.001 and **** p ≤ 0.0001. Group sizes were *n* = 5 treated females, *n* = 6 non-treated females, *n* = 4 treated males and *n* = 5 non-treated males.

**Supplementary Table 4: *Sost*^-/-^ tibial dynamic histomorphometry data (LGK974 and vehicle treated)**

| **Tissue parameters** | **Sex** | **Group** | **Mean** | **±** | **SD** | **Percentage change (%)** | |
| --- | --- | --- | --- | --- | --- | --- | --- |
| **Tibial cortical bone (alizarin red)** |  |  |  |  |  |  |  |
| MS/BS | Male | Vehicle | 34.44 | ± | 18.0306 | -53.04 |  |
|  |  | LGK974 | 16.17 | ± | 17.5770 |  |  |
| BFR/BS | Male | Vehicle | 1.04 | ± | 0.4564 | -56.45 | * |
|  |  | LGK974 | 0.45 | ± | 0.4695 |  |  |
| MAR | Male | Vehicle | 3.22 | ± | 1.1350 | -28.59 |  |
|  |  | LGK974 | 2.30 | ± | 2.1001 |  |  |
|  |  |  |  |  |  |  |  |
| **Tibial cortical bone (calcein)** |  |  |  |  |  |  |  |
| MS/BS | Male | Vehicle | 33.51 | ± | 31.5012 | -27.12 |  |
|  |  | LGK974 | 24.42 | ± | 28.6636 |  |  |
| BFR/BS | Male | Vehicle | 0.86 | ± | 1.0576 | 45.67 |  |
|  |  | LGK974 | 1.25 | ± | 2.0715 |  |  |
| MAR | Male | Vehicle | 1.85 | ± | 1.0560 | 60.52 |  |
|  |  | LGK974 | 2.98 | ± | 5.3382 |  |  |

| **Tibial trabecular bone (alizarin red)** |  |  |  |  |  |  |  |
| --- | --- | --- | --- | --- | --- | --- | --- |
| MS/BS | Male | Vehicle | 12.14 | ± | 9.3921 | -76.01 | * |
|  |  | LGK974 | 2.91 | ± | 1.7594 |  |  |
| BFR/BS | Male | Vehicle | 0.36 | ± | 0.3270 | -94.00 | ** |
|  |  | LGK974 | 0.02 | ± | 0.0257 |  |  |
| MAR | Male | Vehicle | 2.10 | ± | 1.6687 | -63.99 |  |
|  |  | LGK974 | 0.76 | ± | 0.8498 |  |  |
|  |  |  |  |  |  |  |  |
| **Tibial trabecular bone (calcein)** |  |  |  |  |  |  |  |
| MS/BS | Male | Vehicle | 33.62 | ± | 10.3063 | -72.62 | ** |
|  |  | LGK974 | 9.20 | ± | 10.1508 |  |  |
| BFR/BS | Male | Vehicle | 0.66 | ± | 0.4536 | -35.33 |  |
|  |  | LGK974 | 0.43 | ± | 0.4817 |  |  |
| MAR | Male | Vehicle | 1.78 | ± | 0.9731 | 29.11 |  |
|  |  | LGK974 | 2.29 | ± | 2.5583 |  |  |

Statistical comparisons: * p ≤ 0.05 and ** p ≤ 0.01. Group sizes were *n* = 4 treated males and *n* = 4 non-treated males, with two levels analysed for each tibia.

**Supplementary Table 5: Gene expression in *Sost*^-/-^ lumbar vertebrae (LGK974 and vehicle treated)**

| **Target gene** | **Sex** | **Group** | **Mean** | **±** | **SD** | **Percentage change (%)** | |
| --- | --- | --- | --- | --- | --- | --- | --- |
| Axin2 | Male | Vehicle | 0.16 | ± | 0.0389 | -31.18 | * |
|  |  | LGK974 | 0.11 | ± | 0.0099 |  |  |
|  | Female | Vehicle | 0.13 | ± | 0.0301 | -21.33 |  |
|  |  | LGK974 | 0.10 | ± | 0.0379 |  |  |
| Col1a | Male | Vehicle | 579.99 | ± | 88.8760 | -29.08 |  |
|  |  | LGK974 | 411.31 | ± | 151.2571 |  |  |
|  | Female | Vehicle | 564.17 | ± | 176.6885 | -24.92 |  |
|  |  | LGK974 | 423.59 | ± | 139.7688 |  |  |
| TRAP | Male | Vehicle | 5.80 | ± | 1.3061 | -43.40 | * |
|  |  | LGK974 | 3.28 | ± | 1.1732 |  |  |
|  | Female | Vehicle | 4.93 | ± | 1.6316 | -20.71 |  |
|  |  | LGK974 | 3.91 | ± | 2.1113 |  |  |

Statistical comparisons: * p ≤ 0.05. Group sizes were *n* = 5 treated females, *n* = 5 non-treated females, *n* = 4 treated males and *n* = 5 non-treated males.

|  |  |  |  |  |  |  |  |
| --- | --- | --- | --- | --- | --- | --- | --- |

**References**

1 Orriss, I. R. & Arnett, T. R. Rodent osteoclast cultures. *Methods Mol Biol* **816**, 103-117 (2012). <https://doi.org:10.1007/978-1-61779-415-5_8>

2 Berg, S. *et al.* ilastik: interactive machine learning for (bio)image analysis. *Nature Methods* **16**, 1226-1232 (2019). <https://doi.org:10.1038/s41592-019-0582-9>

3 Davies, B. K. *et al.* A Machine Learning-Based Image Segmentation Method to Quantify In Vitro Osteoclast Culture Endpoints. *Calcif Tissue Int* **113**, 437-448 (2023). <https://doi.org:10.1007/s00223-023-01121-z>

4 Schindelin, J. *et al.* Fiji: an open-source platform for biological-image analysis. *Nature Methods* **9**, 676-682 (2012). <https://doi.org:10.1038/nmeth.2019>

5 Chen, J., Yoon, S.-H., Grynpas, M. D. & Mitchell, J. Pre-treatment with Pamidronate Improves Bone Mechanical Properties in Mdx Mice Treated with Glucocorticoids. *Calcif Tissue Int* **104**, 182-192 (2019). <https://doi.org:10.1007/s00223-018-0482-5>

6 De Souza, R. L. *et al.* Non-invasive axial loading of mouse tibiae increases cortical bone formation and modifies trabecular organization: A new model to study cortical and cancellous compartments in a single loaded element. *Bone* **37**, 810-818 (2005). <https://doi.org:https://doi.org/10.1016/j.bone.2005.07.022>
